# Deciphering Cell Type Abundance in Proteomics Data Through Graph Neural Networks

**DOI:** 10.1002/advs.202502987

**Published:** 2025-06-20

**Authors:** Zhiming Dai, Yujie Song, Tuoshi Qi, Hongyu Zhang, Huiying Zhao, Zheng Wang, Yuedong Yang, Yuansong Zeng

**Affiliations:** ^1^ School of Big Data and Software Engineering Chongqing University Chongqing 400000 China; ^2^ School of Computer Science and Engineering Sun Yat‐sen University Guangzhou 510000 China; ^3^ Department of Pathology, Department of Medical Research Center, Sun Yat‐Sen Memorial Hospital Sun Yat‐Sen University Guangzhou 510120 China; ^4^ Jinfeng Laboratory Chongqing China

**Keywords:** graph neural networks, proteomics deconvolution, spatial proteomics

## Abstract

Recent advancements in proteomics sequencing have significantly enhanced our ability to explore cell‐type‐specific signatures within complex tissues, providing critical insights into disease mechanisms. However, current proteomic technologies often suffer from low resolution, resulting in the mixing of multiple cell types during profiling. To address this limitation, cell‐type deconvolution methods are developed to infer cellular composition from proteomic data. While most existing deconvolution methods are focused on transcriptomics, their application to proteomics is hindered by the weak correlation and divergent quantification between transcriptome and proteome data. Although a few proteomic‐specific deconvolution methods are recently emerged, they still exhibit limited capability and performance, partly because they only extract shared information from individual samples while ignoring higher‐order relationships between them. Here, **GraphDEC** is proposed, a novel **graph** neural network‐based method for **dec**iphering cell type proportions in proteomic profiling data. GraphDEC begins by simulating bulk samples from single‐cell proteomic data to create reference data, which is then used to infer cell types in target datasets. Specifically, GraphDEC employs an autoencoder to extract low‐dimensional representations from both reference and target proteomic data, enabling the construction of similarity relationships among samples. These relationships, combined with proteomic data, are processed by a graph neural network that integrates a multi‐channel mechanism and a hybrid neighborhood‐aware approach to learn highly effective representations. To optimize the model, GraphDEC utilizes multiple loss functions, including triplet loss, domain adaptation loss, and Mean Squared Error (MSE) loss, ensuring robust performance and mitigating batch effects. Benchmark experiments demonstrate that GraphDEC achieves state‐of‐the‐art performance across diverse synthetic proteomic datasets from different sequencing technologies and real‐world spatial proteomic datasets. Furthermore, GraphDEC exhibits strong generalization capabilities, showing high efficiency when applied to cross‐species proteomic data and even transcriptomics.

## Graphical abstract

GraphDEC constructs pseudo bulk reference data using a mixup strategy, then learns low dimensional representations via autoencoders to model intra and inter dataset relationships. These are processed through a multi view adaptive neighborhood aware GNN, integrating reference, target, and joint graphs. Multi layer embeddings are fused with learned weights and optimized using MSE, triplet, and domain adaptation losses.

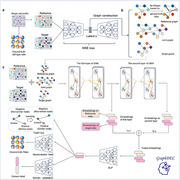

## 1 Introduction

Bulk proteomics plays a pivotal role in studying functional mechanisms and identifying disease biomarkers through the quantitative analysis of protein expression and modifications.^[^ ^1^ ^]^ However, it measures the average protein abundance across entire tissues or organs, thereby masking cellular heterogeneity. Recently, spatial protein omics technologies have emerged, enabling the analysis of protein abundance while preserving spatial context without the need for cell suspension preparation.^[^ ^2^ ^]^ Despite their advantages, spatial omics methods operate at a multicellular resolution, typically capturing data from multiple cells per spatial point, which limits their ability to fully resolve single‐cell heterogeneity.^[^ ^3^ ^]^ In this regard, spatial protein omics retains some characteristics of bulk‐level analysis, as it provides averaged signals across small‐cell populations rather than true single‐cell resolution. In contrast, single‐cell proteomics offers a powerful approach to dissect cellular heterogeneity at the individual cell level.^[^ ^4^ ^]^ However, single‐cell omics in large‐scale studies, particularly in tumor microenvironments, are hindered by high costs and incompatibility with formalin‐fixed or paraffin‐embedded samples. To bridge this gap, deconvolution methods have been developed, leveraging single‐cell data as a reference to infer cell types and proportions from both bulk and spatial samples.^[^ ^5^, ^6^, ^7^ ^]^ These methods provide a cost‐effective and practical solution for studying tissue composition and supporting disease diagnosis, particularly in scenarios where single‐cell resolution is not feasible.

Early efforts in the field primarily focused on the deconvolution of transcriptomic data to infer cell types, laying the groundwork for subsequent methodological advancements.^[^ ^8^ ^]^ These methods can be broadly categorized into five groups based on their computational approaches: probabilistic‐based methods, non‐negative matrix factorization (NMF)‐based methods, graph‐based methods, optimal transport (OT)‐based methods, and deep learning‐based methods. Probabilistic‐based methods are the most widely used for transcriptomics data deconvolution.^[^ ^9^, ^10^, ^11^, ^12^, ^13^, ^14^, ^15^ ^]^ For example, BayesPrism^[^ ^15^ ^]^ integrates single‐cell transcriptomics data from patient‐derived sources as prior information, employing Bayesian algorithms to model the prior distribution and infer the joint posterior distribution of cell type proportions. NMF‐based methods^[^ ^16^, ^17^, ^18^ ^]^ leverage non‐negative matrix factorization (NMF) to extract low‐dimensional features and infer cell types without distributional assumptions. For instance, SPOTlight^[^ ^17^ ^]^ applies seeded NMF to integrate single‐cell RNA‐seq data, decomposing spatial transcriptomics data into cell‐type‐specific profiles and their proportions for accurate spatial mapping.

In contrast to the linear modeling approaches of the aforementioned methods, deep learning‐based methods^[^ ^6^, ^19^ ^]^ have been developed to capture complex non‐linear relationships. For example, Tangram^[^ ^19^ ^]^ maps single‐cell gene expression data to spatial transcriptomics data by representing spatial cells as weighted sums of single cells, enabling both cell‐type deconvolution and downstream spatial analysis tasks. Graph‐based methods,^[^ ^20^, ^21^ ^]^ such as DSTG and *SD* ^2^, utilize graph structures to model spatial relationships and cellular interactions. For instance, DSTG^[^ ^20^ ^]^ employs graph neural networks to construct a cell‐cell interaction graph based on spatial proximity and gene expression similarity, enabling robust deconvolution while preserving spatial context and capturing complex cellular relationships. Although the aforementioned deconvolution methods have achieved advanced performance on transcriptomic data, their application to proteomics has been hindered by the weak correlation and divergent quantification between transcriptome and proteome data. Specifically, protein abundance and transcript expression exhibit distinct distributions and value ranges, and most existing deconvolution methods for transcriptomic data rely on assumed distributions, such as Poisson or negative binomial distributions, which are recognized as unsuitable for proteomic data.

With the advancement of single‐cell proteomics technologies, such as cytometry by time of flight (CyTOF) and fluorescence‐activated cell sorting (FACS)‐based proteomics, an increasing number of single‐cell proteomic profiles have been generated. These datasets hold great potential for inferring cell type ratios in bulk proteomic data when used as references. However, single‐cell proteomic data face several challenges, including significant background noise, poor data quality with variability across runs or technologies, and limited proteome coverage. Additionally, batch effects further complicate analyses in both single‐cell and bulk proteomic data. To address these challenges, scpDeconv^[^ ^22^ ^]^ has been developed as a specialized model for proteomics data. It employs an autoencoder to impute missing protein expression values, enhancing referenceable protein quantities, and a deconvolution head with domain adaptation loss to predict cell type abundance while addressing dataset‐specific distribution differences. While scpDeconv is valuable in various settings, it still exhibits limited capability and performance, partly because it only extracts shared information from individual samples but ignores higher‐order relationships between them. Such topological relationships can be effectively captured by Graph Neural Networks (GNNs), which have demonstrated success in single‐cell and spatial transcriptomic analyses,^[^ ^20^, ^21^, ^23^, ^24^ ^]^ enabling the learning of high‐order representations and improving performance.

Here, we propose GraphDEC, a novel graph neural network‐based deconvolution model designed to accurately predict cell type abundance in bulk and spatial proteomics data. GraphDEC begins by simulating bulk samples from single‐cell proteomic data to create reference data, which is then used to infer cell types in target datasets. GraphDEC then employs an autoencoder to extract low‐dimensional representations from both reference and target proteomic data, enabling the construction of similarity relationships among samples within and between reference and target proteomic data. These relationships, combined with proteomic data, are processed by a graph neural network that integrates a multi‐channel mechanism and a hybrid neighborhood‐aware approach to learn highly effective representations. Finally, the model GraphDEC is optimized using a combination of triplet loss, mean squared error (MSE) loss, and domain adaptation loss. Comprehensive experiments demonstrate that GraphDEC outperforms competing deconvolution methods across diverse datasets, including proteomics data, cross‐species data, transcriptomics data, and real‐world spatial proteomics data. In summary, our method is highly effective for deconvoluting proteomics data and can be extended to other omics data types.

## 2 Results

### 2.1 Overview of GraphDEC

**Figure 1:**
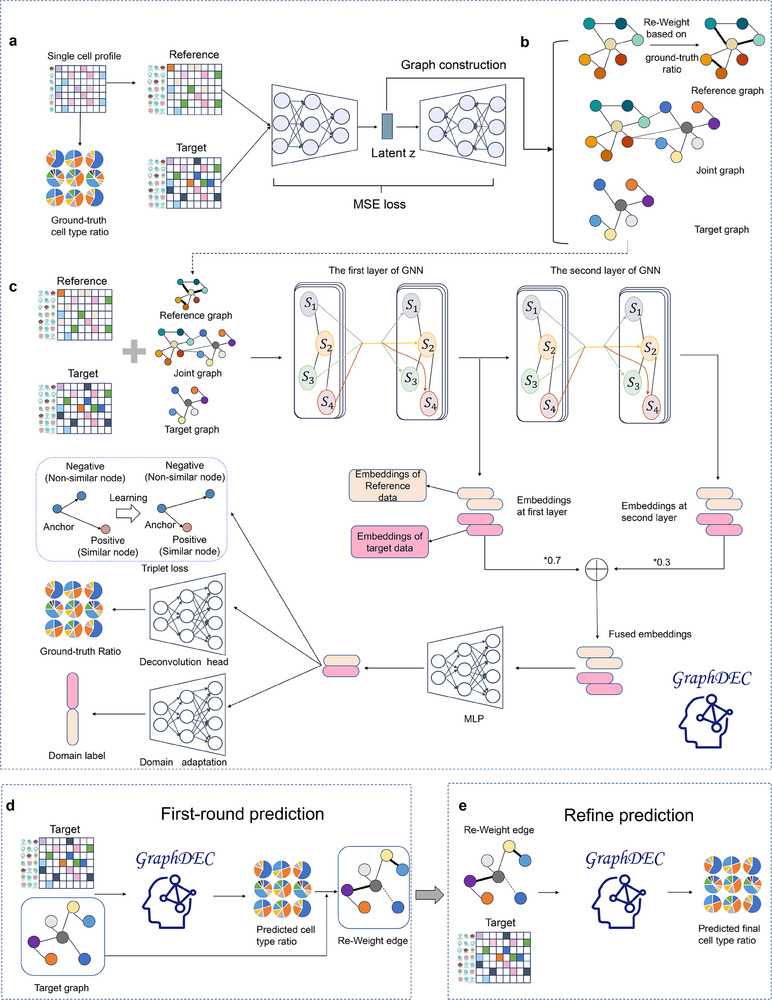
The model architecture of GraphDEC. (a) GraphDEC first constructs pseudo‐bulk reference data from single‐cell proteomics data with cell type labels using a mixup strategy. (b) The constructed reference data and the target data requiring deconvolution are fed into an autoencoder to learn low‐dimensional representations, which are used to establish relationships of samples within and between the reference and target data. The sample relationships in the reference graph are re‐weighted based on the similarity of cell type labels among samples within the reference data. (c) The reference and target data, along with the reference graph, target graph, and joint graph, are iteratively processed by a multi‐view adaptive neighborhood‐aware GNN model and optimized using MSE loss, triplet loss, and domain adaptation loss. Beige and pink represent embeddings from the reference and target datasets, respectively, at the first and second GNN layers. The values ‘*0.7’ and ‘*0.3’ indicate the fusion weights assigned to the first and second layer embeddings, respectively, enabling GraphDEC to integrate multi‐layer features for enhanced representation. (d) The trained GraphDEC model is then utilized to predict cell‐type compositions. These predicted cell type proportions are further employed to re‐weight the edge weights of the target graph. (e) The re‐weighted target graph and target data are jointly fed into GraphDEC to predict the final cell type abundance.

Cell type deconvolution is a computational method for determining cell type proportions from bulk sequencing data, frequently used to analyze heterogeneous cell populations in tumor tissue samples. In this study, we propose GraphDEC, a novel method for deciphering cell type abundance in spatial and bulk proteomic data by leveraging single‐cell proteomic reference data. As shown in **Figure** **1**, GraphDEC consists of four stages.

In Stage 1 of GraphDEC (Figure 1a,b), we collect single‐cell proteomics datasets with ground truth cell types from tissues similar to those in the spatial/bulk proteomic data to be analyzed (referred to as the target data). We then randomly mix single‐cell proteomic data with random cell‐type proportions to construct pseudo‐bulk data as reference data. Each sample in the pseudo‐bulk data, termed a pseudo‐sample, contains multiple cell types, and its proteomic expression is obtained by averaging the expression of its constituent cell types. After data preparation, GraphDEC applies an autoencoder to encode the reference and target data into a low‐dimensional space, obtaining representations. These learned representations are used to construct higher‐order relationships among samples within and between the reference and target data through cosine similarity. Additionally, inspired by the study,^[^ ^25^ ^]^ we compute weights for homophilic edges based on the ground truth cell type ratios in the reference topology graph, assigning larger weights to edges with stronger homophily. This redefined graph helps alleviate the miscalibration problem of GNNs.

In Stage 2 of GraphDEC (Figure 1c), for accurate cell type abundance prediction, GraphDEC employs a Graph Neural Network (GNN) to learn efficient representations of each sample (nodes in the graph) within the reference and target data through a message propagation mechanism. To capture neighborhood information from both global and local perspectives, we incorporate an adaptive neighborhood awareness mechanism by integrating the features of different layers in the GNN model. To perceive neighborhoods from multiple perspectives, GraphDEC utilizes multi‐channel techniques, allowing each channel to observe the same graph from a unique viewpoint. The learned representations from the GNN are passed through a domain discriminator for generative adversarial training, facilitating the removal of batch effects between the reference and target data. Simultaneously, we construct sample triplets to bring the low‐dimensional encodings of anchor cells closer to positive sample cells and farther from negative sample cells. Additionally, a deconvolution head is employed to predict cell‐type proportions from the efficient sample representations.

In Stage 3 of GraphDEC (Figure 1d), the cell‐type proportions of the target proteomic samples are estimated using the trained GraphDEC from Stage 2. To enhance performance, we re‐assign the edge weights of target data based on the input target graph and the predicted cell‐type ratios, as done in Stage 1.

Finally, in Stage 4 of GraphDEC (Figure 1e), we predict the cell type proportions of the target proteomic samples using the trained GraphDEC and the redefined input graph. The output of GraphDEC can be utilized for re‐analyzing the biological mechanisms or tumor microenvironment of specific tissue samples.

### 2.2 Cell Type Ratio Inference for Proteomics Data

To assess the performance of GraphDEC in cell‐type deconvolution, we rigorously evaluated our method using a comprehensive benchmark comprising seven reference‐target proteomics datasets. These datasets were derived from seven distinct single‐cell proteomics cases, each encompassing multiple single‐cell datasets. The cases represented a diverse range of tissues, sequencing technologies, species, and sources (detailed information is available in the data availability section). For each reference‐target dataset, we followed a systematic approach to evaluate deconvolution accuracy. First, we selected one or more single‐cell proteomic datasets to generate pseudo‐bulk proteomic data with known cell‐type compositions, serving as the reference data. Subsequently, we utilized the remaining single‐cell proteomic data within the same case to simulate pseudo‐bulk proteomic data, which served as the target data. Once the reference and target datasets were constructed, we applied GraphDEC to predict the cell proportions in the corresponding target data. To benchmark GraphDEC's performance, we compared it against three state‐of‐the‐art deconvolution methods originally developed for transcriptomic data‐Scaden,^[^ ^6^ ^]^ DSTG,^[^ ^20^ ^]^ CellDART,^[^ ^26^ ^]^ Scanorama,^[^ ^27^ ^]^ AutoGeneS,^[^ ^28^ ^]^ DestVI,^[^ ^11^ ^]^ and Tangram^[^ ^19^ ^]^‐ as well as the latest proteomics deconvolution method, scpDeconv.^[^ ^22^ ^]^ The evaluation metrics included Lin's concordance correlation coefficient (CCC), root mean square error (RMSE), and Pearson correlation coefficient (PCC), ensuring a robust and comprehensive assessment of each method's accuracy and reliability. The Pearson correlation coefficient and Lin's concordance correlation coefficient were calculated by comparing the model's predicted cell abundance values with the corresponding gold standard values for each sample in the target dataset.

**Figure 2:**
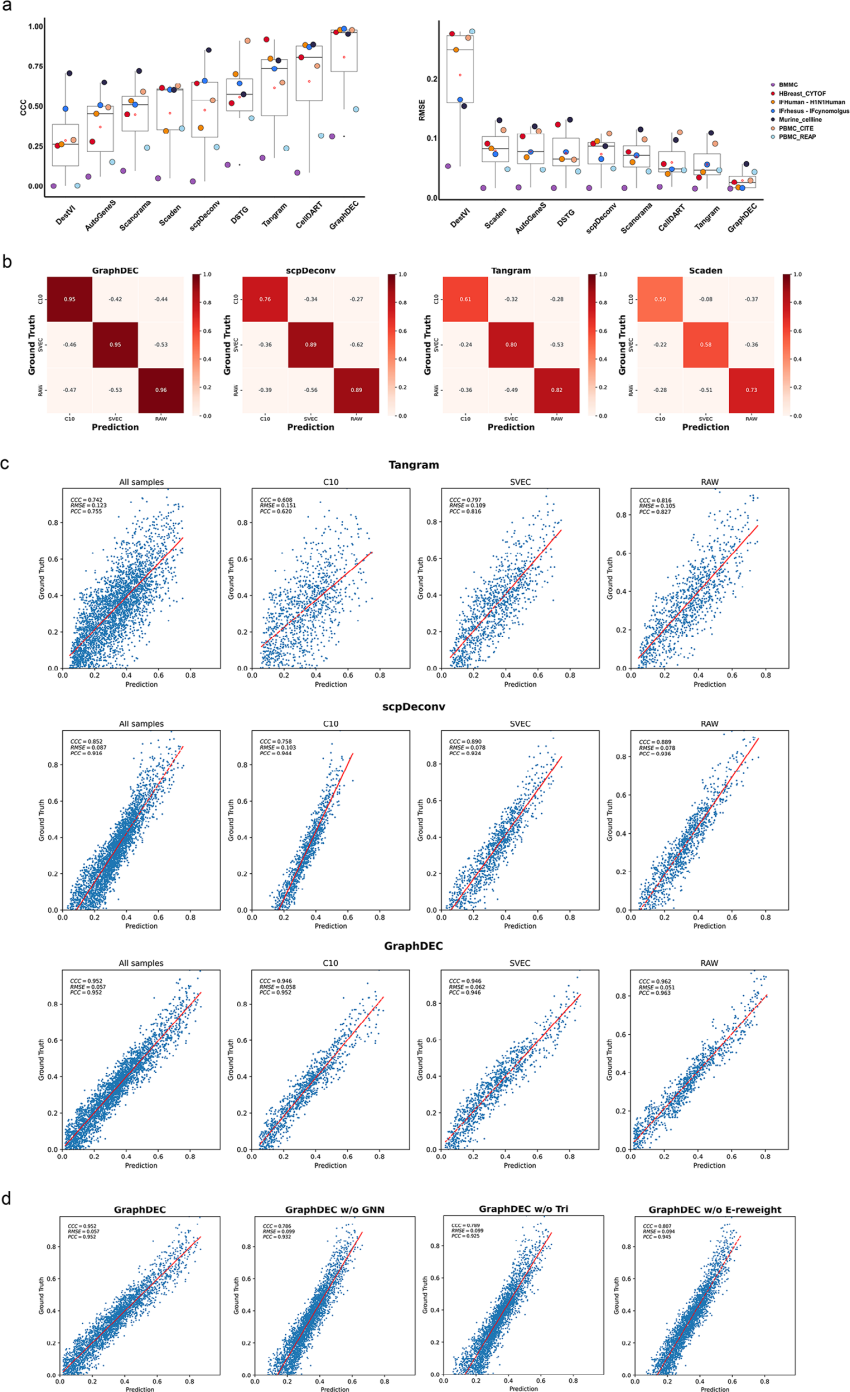
The performance of GraphDEC inferring cell type ratio for proteomics data. (a) Boxplots showed CCC and RMSE values for each method. The boxplots are defined as follows: the minimum is calculated as the 25th percentile minus 1.5 times the interquartile range (IQR), and the maximum is calculated as the 75th percentile plus 1.5 times the IQR. The hinges of the box represent the interquartile range (IQR), while the whiskers extend to 1.5 times the IQR. The center line of the boxplot indicates the median and the bounds of the box correspond to the 25th and 75th percentiles. An open red circle denotes the average values within each boxplot, and black dots represent outliers. This analysis is based on n = 7 biologically independent reference‐target proteomics datasets. The x‐axis labels the different deconvolution methods, and the y‐axis represents the measured values. (b) The confusion matrices of the prediction results from each method on the Murine_cellline dataset. The diagonal cells represent the CCC values between the predicted and true values for each specific cell type. (c) Scatter plots of the ground truth (y‐axis) and predicted (x‐axis) proportions of three cell types (C10, SVEC, and RAW) in the case of Murine cell line datasets using Tangram, scpDeconv, and GraphDEC, respectively. (d) The performance of GraphDEC when removing the Graph Network neural(GNN), triple loss (Tri), and edge re‐weight mechanism (E‐reweight).

As shown in **Figure** **2**a, our method demonstrated superior overall performance across all datasets. Specifically, GraphDEC achieved an average CCC score of 80.7%, representing a 15% improvement over the next best method (CellDART, with an average CCC = 65.7%). In terms of RMSE, GraphDEC achieved a score of 0.029, surpassing the closest competitor, Tangram, which had an RMSE of 0.056. These results highlight GraphDEC's ability to provide more accurate predictions, with cell type proportions that align more closely with the ground truth. Interestingly, we observed that the proteomics‐based method, scpDeconv, underperformed compared to Tangram, a method originally designed for transcriptomic data. This discrepancy may stem from Tangram's flexibility, as it does not rely on strict data distribution assumptions and is designed to be a general‐purpose tool for diverse omics data types. Scaden and scpDeconv exhibited comparable performance in terms of average CCC and RMSE, respectively. In contrast, DestVI, which relies on stringent assumptions about transcript distribution in transcriptomic data, showed the poorest performance when applied to cross‐source reference and target data, making it unsuitable for proteomics applications. Compared to the baseline methods DSTG, AutoGeneS, and Scanorama, GraphDEC consistently achieved the highest average CCC across proteomics datasets. Additionally, a similar performance trend was observed when evaluating methods using PCC (Figure S3a, Supporting Information).

All methods struggled to perform well on the BMMC and PBMC_REAP datasets. Thus, we re‐evaluated GraphDEC by restricting the analysis to Monocytes, T cells, and B cells in PBMC_REAP. As shown in Figure S1 (Supporting Information), the results revealed that GraphDEC achieved a CCC increase to 96.2%, indicating robust performance on these well‐defined cell types. While other methods also showed improvements, GraphDEC consistently outperformed them. These results suggest that the low performance observed in the PBMC_REAP dataset may not be primarily due to batch effects but rather biological variation. In contrast to the clear separation of the three analyzed cell types, many of the original cell types were highly mixed (Figure S2, Supporting Information). For example, Pre‐B_cell_CD34‐ cells were mixed with T cells and Monocytes, and NK cells were mixed with Monocytes, T, and B cells. Furthermore, most of the remaining cell types had very low abundance, which may also have limited model performance.

To further validate the superior performance of GraphDEC, we re‐analyzed its performance on the Murine_cellline case dataset, which contains three shared cell types generated by different single‐cell proteomic technologies (N2 and nanoPOTS). These datasets consist of three common murine cell lines (C10, RAW, and SVEC). As shown in the confusion matrices (Figure 2b; Figure S3b, Supporting Information), GraphDEC accurately predicted the cell type proportions, with CCC values exceeding 95% for each cell type. In contrast, the CCC values for other competing methods remained below 90% for all cell types. These results were further supported by scatter plots (Figure 2c; Figure S3c,d, Supporting Information), which illustrated the true (y‐axis) versus predicted (x‐axis) proportions for each cell type (C10, SVEC, and RAW) in the Murine_cellline case dataset across all methods. A similar trend was observed for the HBreast_CYTOF dataset, as shown in Figure S4 (Supporting Information). The removal of the GNN component resulted in a significant performance drop, with an average CCC value decreasing by 15% (2d). The removal of the triplet loss and edge re‐weighting mechanism also led to a slight decrease in CCC values. Additionally, we investigated the impact of the number of single cells in each pseudo‐sample on GraphDEC's performance. As shown in Figure S5 (Supporting Information), the results demonstrated that GraphDEC's performance was only slightly affected by the number of mixed cells in each pseudo‐sample, and it consistently outperformed competing methods across all scenarios. In summary, GraphDEC achieved robust and reliable performance on the proteomics datasets, demonstrating its effectiveness in cell‐type deconvolution tasks.

To evaluate the protein marker dependency of each method, we conducted ablation studies by removing the top 1 and top 5 differentially expressed proteins per cell type. As shown in Figure S6 (Supporting Information), GraphDEC showed minimal performance drop (0.1% and 6%, respectively), outperforming Scaden (5%, 10.4%) and Tangram (4%, 19.2%). These results suggest that GraphDEC captures broad protein expression patterns rather than relying solely on cell type‐specific markers. To evaluate the contributions of key components in GraphDEC, we conducted ablation studies on the HBreast_CYTOF proteomics dataset. As shown in Figure S7 (Supporting Information), excluding the GNN caused a 22.4% drop in average CCC, and removing the triplet loss and E‐reweight modules led to slight declines. These results confirm that the full integration of all components is essential for maximizing GraphDEC's accuracy and robustness. To assess the robustness of GraphDEC, we conducted additional experiments on the case dataset, evaluating the impact of key hyperparameters, including the random seed, embedding size, α, epoch, and learning rate (lr). As shown in Figure S7 (Supporting Information), variations in these hyperparameters had minimal impact on the model's performance, indicating that GraphDEC remains stable across different settings. These findings further demonstrate the robustness of our approach. To evaluate scalability, we measured runtime and memory usage on datasets ranging from 1000 to 20,000 spots. As shown in Figure S8 (Supporting Information), GraphDEC maintained reasonable computational demands, showing near‐linear memory scaling and runtime comparable to scpDeconv, outperforming DestVI and slightly trailing Tangram. These results confirm GraphDEC's practicality for large‐scale applications while preserving strong performance.

### 2.3 Cross‐Species Proteomics Deconvolution Performance

**Figure 3:**
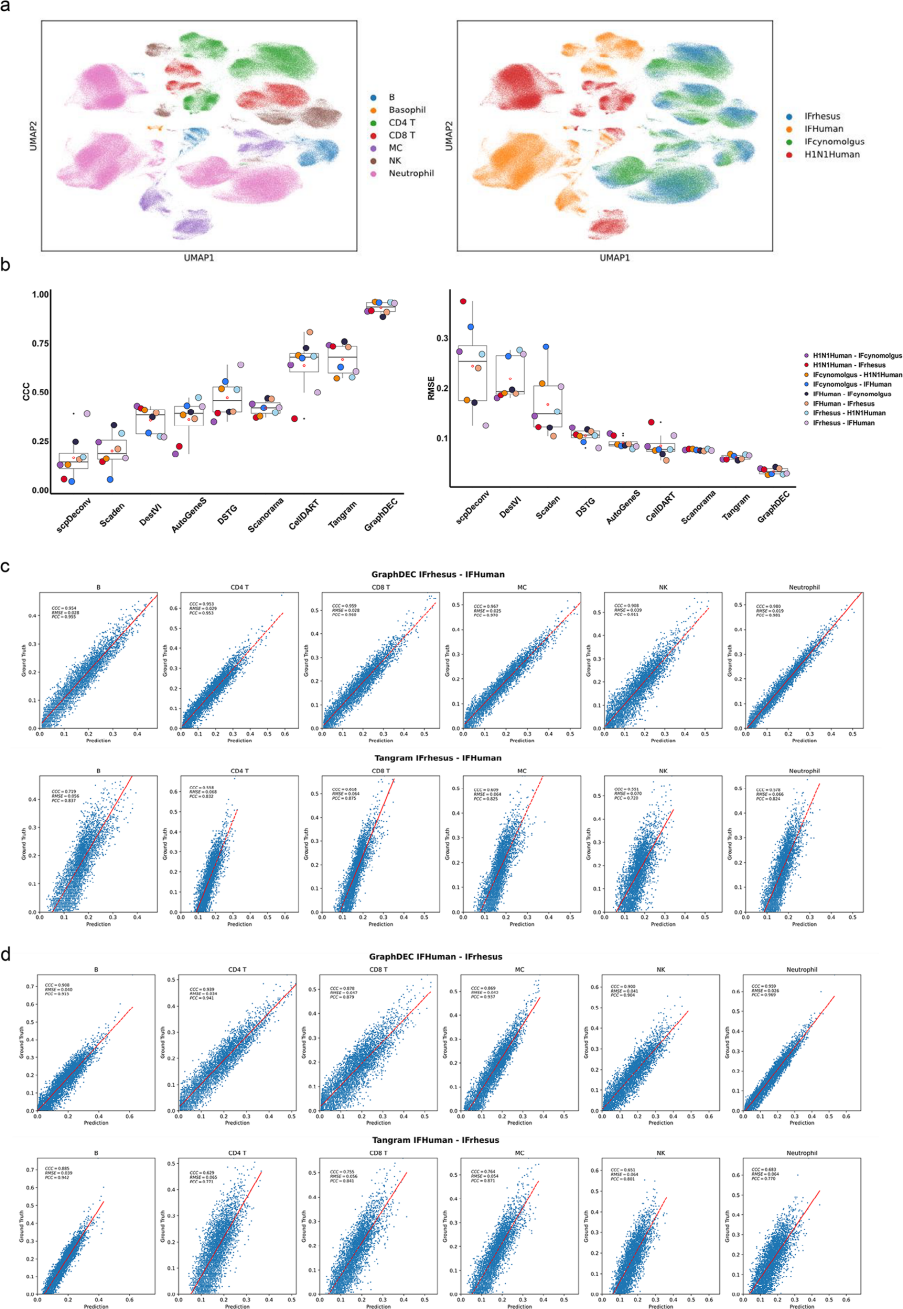
The performance of GraphDEC on the cross‐species proteomics datasets. (a) UMAP scatter plot of the single‐cell cross‐species proteomics data used for constructing reference data, which are colored by cell types and species. (b) Boxplots showed CCC and RMSE values for each method. The boxplots are defined as follows: the minimum is calculated as the 25th percentile minus 1.5 times the interquartile range (IQR), and the maximum is calculated as the 75th percentile plus 1.5 times the IQR. The hinges of the box represent the interquartile range (IQR), while the whiskers extend to 1.5 times the IQR. The center line of the boxplot indicates the median and the bounds of the box correspond to the 25th and 75th percentiles. An open red circle denotes the average values within each boxplot, and black dots represent outliers. This analysis is based on n = 8 biologically independent reference‐target cross‐species proteomics datasets. The x‐axis labels the different deconvolution methods, and the y‐axis represents the measured values. (c) Scatter plots of the ground truth (y‐axis) and predicted (x‐axis) proportions of three cell types (B, CD4 T, CD8 T, MC, NK, and Neutrophil) in the case of IFrhesus‐IFHuman datasets using GraphDEC and Tangram, respectively. (d) Scatter plots of the ground truth (y‐axis) and predicted (x‐axis) proportions of three cell types (B, CD4 T, CD8 T, MC, NK, and Neutrophil) in the case of IFHuman‐IFrhesus datasets using GraphDEC and Tangram, respectively.

To investigate the performance of GraphDEC in deconvoluting proteomics data across species, we applied GraphDEC to proteomics data from humans and monkeys. For evaluation, we utilized four publicly available single‐cell proteomics datasets, each containing 120,000 blood cells categorized into seven cell types. These datasets were obtained from (i) healthy humans challenged with the H1N1 virus (termed H1N1Human), (ii) humans stimulated with IFNγ (termed IFHuman), (iii) rhesus macaques stimulated with IFNγ (termed IFrhesus), and (iv) cynomolgus monkeys stimulated with IFNγ (termed IFcynomolgus). These four single‐cell proteomics datasets were used to construct eight synthetic cross‐species reference‐target bulk proteomics datasets. Compared to single‐cell proteomics datasets from the same species, deconvolution for cross‐species proteomics datasets proved more challenging due to significant batch effects. For instance, cells from humans and monkeys exhibited distinct boundaries, and cells of each cell type from humans and monkeys did not cluster together in the UMAP visualization results, as shown in **Figure** **3**a. Despite the substantial batch effects in cross‐species datasets, GraphDEC consistently achieved the best performance compared to competing methods (Figure 3b). Specifically, GraphDEC achieved an average CCC value of 93% across the eight cross‐species pseudo‐bulk reference‐target proteomics datasets, representing a 26.4% improvement over the second‐best method, Tangram. GraphDEC consistently outperformed the baseline methods DSTG, CellDART, AutoGeneS, and Scanorama, achieving the highest average CCC on cross‐species proteomics datasets and showing a 29.6% gain over CellDART. A similar performance trend was observed when evaluated using RMSE and PCC, as shown in Figure 3b and Figure S9a (Supporting Information), respectively.

GraphDEC achieved a high performance for cross‐species data but performed less effectively on BMMC or PBMC_REAP. This performance gap stems from differences in dataset complexity: BMMC or PBMC_REAP contains mixed cell types and imbalanced cell type distributions, whereas cross‐species data features well‐separated major cell types with stronger biological signals and more balanced distributions. GraphDEC effectively addresses cross‐species batch effects through its GNN architecture and triplet loss mechanism, but struggles with the complex mixed populations and imbalances in BMMC or PBMC_REAP. These results underscore the significant impact of biological variation and dataset composition on the performance of cell‐type deconvolution methods.

To further investigate the rationale behind the superior performance of GraphDEC, we re‐analyzed two cases: IFrhesus‐IFHuman and IFHuman‐IFrhesus. First, we visualized the abundance of proteins in human and monkey samples using a heatmap and plotted the raw data from IFrhesus and IFHuman, as shown in Figure S9b (Supporting Information). The visualization results revealed distinct distributions of key proteins between IFrhesus and IFHuman. This phenomenon was further corroborated by UMAP visualization (Figure S9c, Supporting Information), where the two datasets exhibited clear separation, and the same cell types from these datasets did not merge. These results highlight the presence of significant batch effects in the raw data. As shown in the scatter plots (Figure 3c,d), GraphDEC accurately predicted each cell type and consistently outperformed competing methods in both cases (IFrhesus‐IFHuman and IFHuman‐IFrhesus). These findings demonstrate that GraphDEC effectively mitigates batch effects between human and monkey datasets and learns informative low‐dimensional representations. In summary, GraphDEC is highly effective for deconvoluting proteomics datasets across species, even in the presence of batch effects.

### 2.4 GraphDEC Enables Deconvoluting for Transcriptomics Data

In this section, GraphDEC was applied to transcriptomics data to demonstrate its generalizability across omics modalities. To ensure robust feature selection, the top 500 genes were chosen based on variance. No specific modifications for GraphDEC were required, as the method does not rely on assumptions about data distribution (e.g., Poisson or Negative Binomial models commonly used for transcriptomics). Instead, GraphDEC leverages its GNN architecture to learn low‐dimensional representations and propagate information between similar spots, which helps to alleviate issues such as dropout events and gene length bias. By capturing global patterns in the data, GraphDEC effectively overcomes transcriptomics‐specific challenges.

As suggested in the previous study,^[^ ^6^ ^]^ we conducted cell type composition analysis on transcriptomics data from three tissues: Mouse Brain, Pancreas, and PBMC. We utilized the preprocessed and constructed reference‐target datasets provided by the previous method^[^ ^6^ ^]^ for these analyses. Specifically, the Mouse Brain dataset contained 24,000 reference samples and 6000 target samples, with seven shared cell types and 14,155 common genes. The Pancreas dataset included 26,000 reference samples and 2000 target samples, with eight shared cell types and 20,938 common genes. The PBMC dataset consisted of 24,032 reference samples and 8000 target samples, with six shared cell types and 11,328 common genes. In this evaluation, we compared GraphDEC with seven state‐of‐the‐art methods: Cell2location,^[^ ^10^ ^]^ Stereoscope,^[^ ^13^ ^]^ scpDeconv, Tangram, Scaden, DSTG, CellDART, Scanorama, AutoGeneS, DestVI, and CIBERSORTx.^[^ ^5^ ^]^ Notably, Cell2location, Stereoscope, and CIBERSORTx were not compared on proteomics datasets due to their reliance on rigorous distribution assumptions, which are incompatible with proteomics data. All methods were evaluated using three metrics: Lin's Concordance Correlation Coefficient (CCC), Root Mean Square Error (RMSE), and Pearson Correlation Coefficient (PCC).

**Figure 4:**
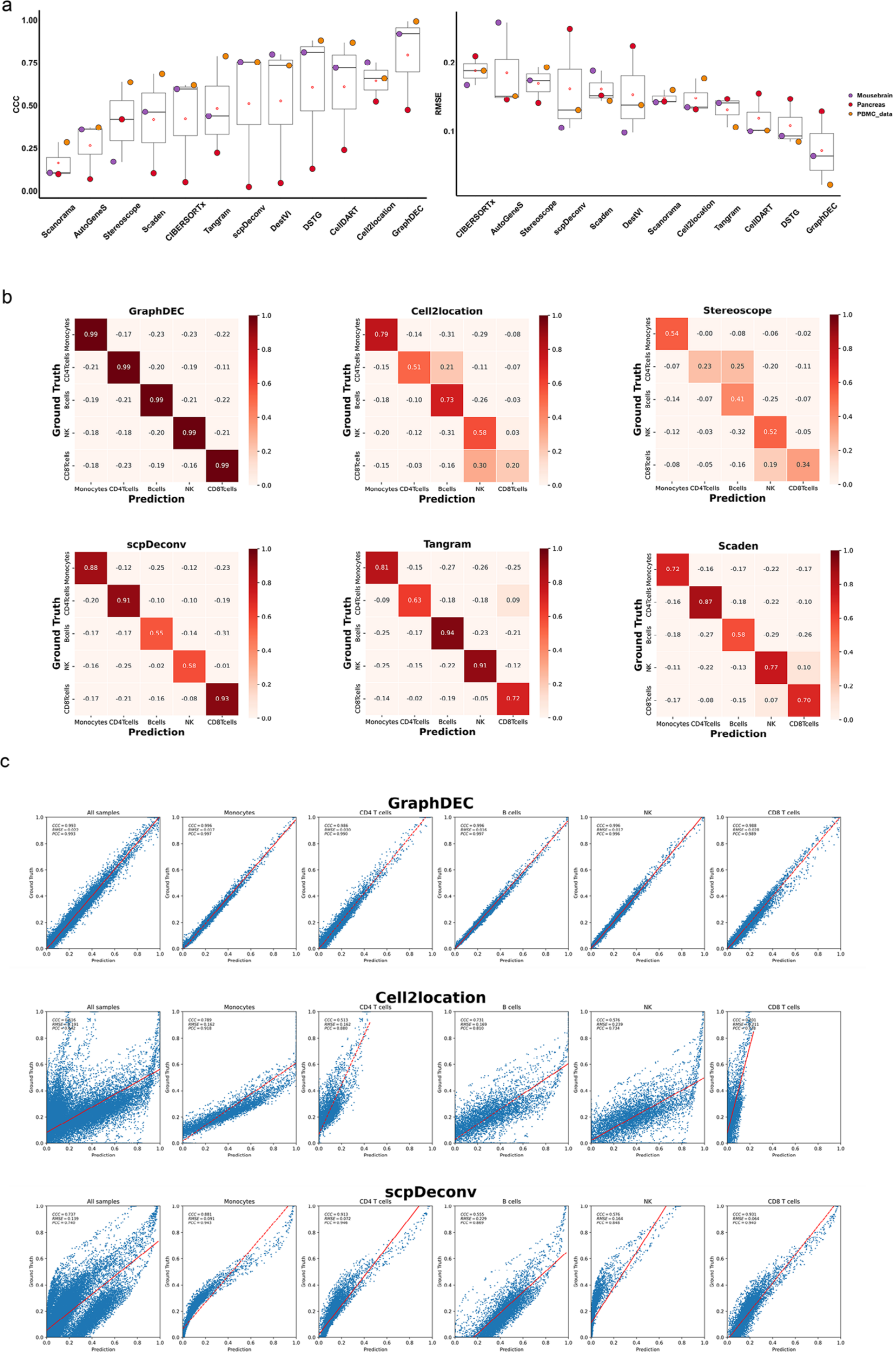
The performance of GraphDEC deconvoluting for transcriptomics data. (a) Boxplots showed CCC and RMSE values for each method. The boxplots are defined as follows: the minimum is calculated as the 25th percentile minus 1.5 times the interquartile range (IQR), and the maximum is calculated as the 75th percentile plus 1.5 times the IQR. The hinges of the box represent the interquartile range (IQR), while the whiskers extend to 1.5 times the IQR. The center line of the boxplot indicates the median and the bounds of the box correspond to the 25th and 75th percentiles. An open red circle denotes the average values within each boxplot, and black dots represent outliers. This analysis is based on n = 3 biologically independent reference‐target transcriptomics datasets. The x‐axis labels the different deconvolution methods, and the y‐axis represents the measured values. (b) The confusion matrices of the prediction results from each method in the case of PBMC_data. The diagonal cells represent the CCC values between the predicted and true values for each specific cell type. (c) Scatter plots of the ground truth (y‐axis) and predicted (x‐axis) proportions of three cell types (Monocytes, CD4 T cells, B cells, NK, and CD8 T cells) in the case of PBMC_data datasets using GraphDEC, Cell2location, and scpDeconv, respectively.

The quantitative results of each method are reported in **Figure** **4**a. Our method, GraphDEC, consistently achieved the best overall performance. Specifically, GraphDEC obtained an average CCC of 79.6%, which was 15% higher than the second‐ranked method, Cell2location. Three deep learning‐based methods—DestVI, scpDeconv, and Tangram—exhibited similar performance. Across all transcriptomics datasets, GraphDEC achieved the highest average CCC compared to the baseline methods DSTG, CellDART, AutoGeneS, and Scanorama, with an 18.6% increase over CellDART. A similar trend was observed when evaluating the methods using other metrics, including RMSE (Figure 4a) and PCC (Figure S10a, Supporting Information). To further validate the superior performance of GraphDEC, we visualized the predicted performance for each cell type on the PBMC dataset using confusion matrices and scatter plots, as shown in Figure 4b,c. GraphDEC consistently achieved the best performance for each cell type. A similar trend was observed for the Mouse Brain dataset, as illustrated in Figures S10b and S11 (Supporting Information). Overall, our method demonstrates high efficiency for transcriptomics deconvolution tasks.

To further evaluate the robustness of GraphDEC under dropout‐heavy conditions, we performed benchmark comparisons on two public single‐cell transcriptomic datasets—Human_Optic_Nerve and Gland_Atlas—downloaded from the CELLxGENE repository. For the Human_Optic_Nerve dataset, we selected cranial nerve II samples as the reference and optic disc samples as the target, resulting in a 76.74% dropout rate in the target data. For Gland_Atlas, we used the submandibular gland as reference and the minor salivary gland as target, yielding an 81.53% dropout rate in the target data. To simulate more severe dropout scenarios, we further downsampled the total UMI (unique molecular identifier) counts per spot using the scuttle package, following the protocol used in RCTD. This yielded synthetic datasets with dropout rates of 90.69% for Human_Optic_Nerve and 90.76% for Gland_Atlas. As shown in Figure S12 (Supporting Information), GraphDEC consistently achieved the best performance across all metrics. On the original datasets, it reached an average CCC score of 67.35%, outperforming the second‐best method CellDART by 3.9%. On the downsampled datasets with dropout exceeding 90%, GraphDEC maintained top performance, with a CCC score of 48.25%, exceeding CellDART by 4.35%. While all methods showed some degradation under extreme dropout, GraphDEC demonstrated greater robustness and maintained leading performance across all metrics. These results highlight the effectiveness of the GNN architecture in GraphDEC for mitigating dropout effects in transcriptomic data. Similar performance trends were observed for PCC and RMSE (Figure S12, Supporting Information).

### 2.5 Spatial Proteomics Data Deconvolution Performance

**Figure 5:**
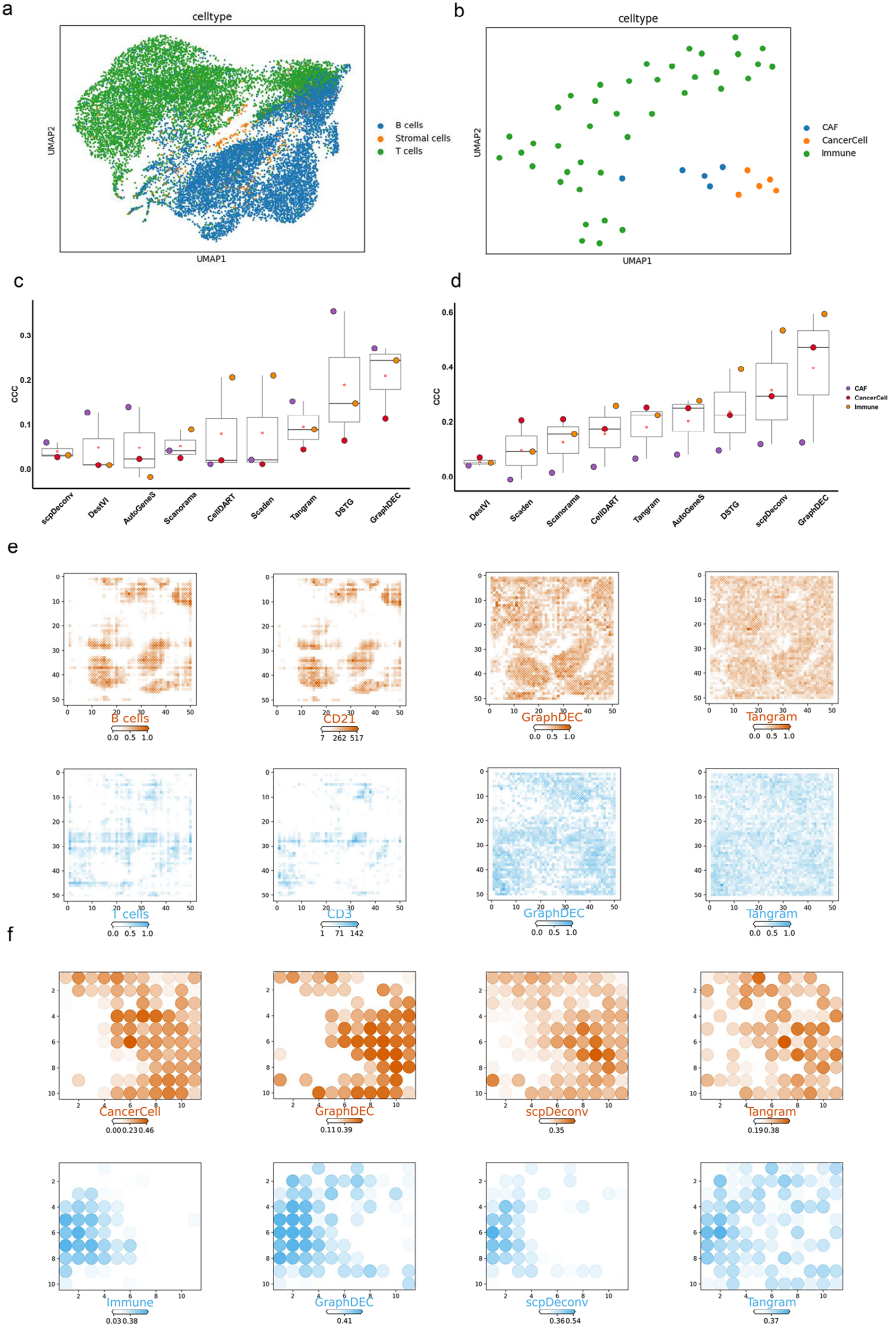
The performance of GraphDEC on spatial proteomics datasets. (a) UMAP visualization of single‐cell spatial proteomics data for the human_palatine_tonsil dataset, with points colored by cell type. (b) UMAP visualization of single‐cell spatial proteomics data for the mouse_PDAC dataset, with points colored by cell type. (c) Boxplots of CCC values for each deconvolution method on the human_palatine_tonsil dataset. The boxplots are defined as follows: the minimum is the 25th percentile minus 1.5 times the interquartile range (IQR), and the maximum is the 75th percentile plus 1.5 times the IQR. The box hinges represent the IQR, while the whiskers extend to 1.5 times the IQR. The center line indicates the median, and the box bounds correspond to the 25th and 75th percentiles. An open red circle denotes the mean value, and black dots represent outliers. This analysis is based on n = 3 cell types. The x‐axis labels the deconvolution methods, and the y‐axis represents the CCC values. (d) Boxplots of CCC values for each deconvolution method on the mouse_PDAC dataset. The boxplot definitions are identical to (c). This analysis is based on n = 3 cell types. (e) Heatmaps showing the spatial abundance of B cells and T cells predicted by GraphDEC and Tangram, respectively, along with marker gene expression for B cells and T cells in the human_palatine_tonsil dataset. The x and y axes represent the true spatial locations in the section as provided by the target Tonsil dataset. (f) Heatmaps showing the spatial abundance of CancerCell and Immune cells predicted by GraphDEC, scpDeconv, and Tangram, respectively, in the mouse_PDAC dataset. The x and y axes represent the true spatial locations in the section as provided by the target PDAC dataset.

To evaluate the performance of GraphDEC on real‐world bulk proteomics data, we applied our method to two spatial proteomics datasets. First, we analyzed the human_palatine_tonsil spatial proteomics data, generated using antibody‐derived DNA tags (ADTs). The reference dataset consisted of 17,367 tonsillar cells, categorized into three major groups: B cells, T cells, and stromal cells, as illustrated in **Figure** **5**a. Next, we examined a pancreatic ductal adenocarcinoma (PDAC) spatial proteomics dataset, obtained through laser microdissection (LMD) coupled with LC‐MS/MS detection. To further dissect the complexity of PDAC, we collected single‐cell‐type proteomics data from five PDAC mice using FACS sorting followed by LC‐MS/MS. This reference dataset included 50 samples and 5788 identified proteins, spanning three major cell lineages: pancreatic cancer cells (CancerCell), cancer‐associated fibroblasts (CAF), and pancreatic immune cells (Immune), as shown in Figure 5b.

The CCC values for real spatial proteomics datasets were calculated using experimentally derived cell type distributions, obtained through marker‐based or antibody‐based techniques. This direct comparison with validated ground truth avoids the need for generating pseudo‐samples in the target domain. As demonstrated in Figure 5c, GraphDEC consistently outperformed competing methods. Specifically, on the human_palatine_tonsil dataset, GraphDEC achieved an average CCC of 21% across all cell types, representing a 2.1% improvement over the second‐ranked method, DSTG. Notably, all methods performed poorly on stromal cells, likely due to their significantly lower abundance compared to the other two cell types. For the PDAC spatial proteomics data, GraphDEC also demonstrated superior performance, achieving an average CCC of 39.7% across the three cell types, which was an 8.13% improvement over baseline methods (Figure 5d). Compared to the baselines, CellDART, AutoGeneS, and Scanorama, GraphDEC consistently achieved the highest average CCC across spatial proteomics datasets

To further validate the predictive performance of GraphDEC, we visualized the cell type with the highest performance in both spatial proteomics datasets using heatmaps. As shown in Figure 5e‐f and Figure S13 (Supporting Information), GraphDEC exhibited the closest alignment with established cell‐type‐specific markers and the ground truth of cell‐type distribution compared to competing methods. These results further highlight the capability of GraphDEC to accurately predict cell type abundance in spatial proteomics data.

GraphDEC yields lower CCC values on spatial proteomics data compared to pseudo‐bulk and cross‐species settings. This likely stems from the limited diversity of cell type proportion patterns in the reference, which, when coupled with the greater compositional complexity of real spatial data, hampers the model's ability to accurately predict unseen spot‐level distributions. In contrast, pseudo‐bulk datasets benefit from consistent generation strategies between reference and target, leading to better alignment and improved performance. These findings highlight GraphDEC's reliance on a comprehensive reference and suggest several future directions, including constructing more diverse reference datasets, developing reference‐independent approaches, or leveraging foundation models to improve generalizability.

## 3 Discussion

GraphDEC is a graph neural network‐based model for cell type deconvolution in bulk and spatial proteomics. It generates pseudo‐bulk references from single‐cell data and extracts low‐dimensional embeddings via an autoencoder. By constructing similarity graphs, GraphDEC captures relationships within and across datasets using a multi‐channel, neighborhood‐aware GNN. Trained with triplet, MSE, and domain adaptation losses, GraphDEC achieves state‐of‐the‐art performance across diverse synthetic and real proteomic datasets.

The GNN‐based formulation in GraphDEC brings unique advantages for proteomics deconvolution. Unlike traditional methods that treat samples independently, our approach constructs a graph structure linking both reference and target samples based on protein expression similarity. This allows the model to capture local and global relationships among samples and effectively propagate information across them. Graph neural networks, particularly GraphSAGE, enhance robustness to noise and improve generalization by aggregating neighborhood information, which is especially valuable for proteomics data characterized by fewer features and smoother expression patterns. By leveraging cross‐sample and cross‐dataset connectivity, GraphDEC can learn more discriminative and stable representations, leading to more accurate estimation of cell‐type proportions. These benefits align with recent successes of GNNs in single‐cell omics tasks, further supporting their application in this context.

Existing several deconvolution methods developed for transcriptomics data often rely on assumptions and input formats that are incompatible with proteomics. Unlike transcriptomic data, proteomics data consist of continuous intensity values and have lower dimensionality, limiting the effectiveness of models designed for high‐resolution gene expression.

GraphDEC's clinical application requires attention to ethical considerations. Biases in non‐representative reference datasets may impact model generalizability, and privacy concerns could arise when proteomic data is combined with other patient information. Future use should ensure diverse cohort validation and strict data anonymization.

Although GraphDEC demonstrates accurate deconvolution performance, it has several limitations. First, GraphDEC relies on single‐cell proteomic data to construct its reference, which may limit its accuracy when the target data includes cell types not represented in the reference. This lack of comprehensive coverage could compromise the precision of deconvolution predictions. Second, GraphDEC currently focuses solely on single‐modal data (proteomics), overlooking the potential advantages of integrating multi‐modal information, such as transcriptomics or imaging data. Incorporating multi‐modal data could provide a more holistic understanding of cellular states and further enhance performance. Finally, GraphDEC does not yet leverage existing large‐scale models, which have shown significant promise in other domains. Future developments should explore integrating such models to improve its predictive power and scalability.

## 4 Experimental Section

GraphDEC is a graph neural network‐based deconvolution method designed to accurately predict cell type abundance in bulk and spatial proteomics data. The framework of GraphDEC consists of two core components: a cell‐type mix‐up module and a multi‐channel graph neural network (GNN). The GNN is optimized through a composite loss function that integrates triplet loss, domain adaptation loss, and mean squared error (MSE) loss. GraphDEC takes as input reference data with annotated cell type labels and target data requiring deconvolution, and it outputs the predicted cell type abundance for each sample in the target data.

### Pseudo‐Single‐Cell Data Mixup

To deconvolve the target bulk proteomics data, we initially constructed pseudo‐reference bulk proteomics data by aggregating single‐cell proteomics data with corresponding cell‐type labels using the mixup data augmentation strategy.^[^ ^29^ ^]^ Specifically, we randomly sampled single cells with known cell types from the training set and applied the mixup technique to their proteomics data, thereby generating pseudo‐bulk proteomics samples with defined cell type abundances. In this process, random combinations of known cell types were selected from the single‐cell reference proteomic data, and the average protein abundance from these combinations was computed to represent the proteome profile of each pseudo‐sample. The cell type ratio for each pseudo‐sample was determined by dividing the count of each cell type by the total number of cell types in the combination. These ratios were then compiled into a vector, encapsulating the cell‐type proportions of the pseudo‐bulk sample. Through this methodology, we established pseudo‐bulk samples for both the synthetic reference and target datasets, denoted as $X^{r}\in R^{N_{r}\times p}$ and $X^{t}\in R^{N_{t}\times p}$, respectively. The ground truth cell labels for the reference data, represented by $Y^{r}\in R^{N_{r}\times n}$, were prepared for subsequent training tasks. Here, *N*_*r*_ and *N*_*t*_ indicate the number of pseudo‐samples in the reference and target proteomics datasets, while *n* and *p* mean the number of distinct cell types and proteins, respectively.

### Autoencoder for Feature Projection

To establish relationships between samples, we compute cosine similarity based on their low‐dimensional representations, which are derived by projecting the protein abundance of reference‐target data using an autoencoder. The AE model is trained exclusively on reference‐target data with shared proteins. Specifically, the encoder of the AE is used to transform the protein expression matrices of the reference‐target data into fixed‐size vector representations. At the *l*‐th encoding layer of the encoder, the output *H* ^(*l*)^ is computed as follows:

(1) $$H^{\left(l\right)}=\phi \left(W_{e}^{\left(l\right)}H^{\left(l-1\right)}+b_{e}^{\left(l\right)}\right),$$

where ϕ denotes the ReLU activation function, and $W_{e}^{\left(l\right)}$ and $b_{e}^{\left(l\right)}$ represent the weight matrix and bias parameters, respectively. We iteratively initialize *H* ^(0)^ as *X* ^*r*^ and *X* ^*t*^ to project the reference and target, respectively. The final layer of the encoder serves as the latent representation *Z*, which is then passed to the decoder of the AE to reconstruct the input matrix. The output of the *m*‐th layer in the decoder is calculated as:

(2) $$H^{\left(m\right)}=\phi \left(W_{d}^{\left(m\right)}H^{\left(m-1\right)}+b_{d}^{\left(m\right)}\right),$$

where ϕ is the ReLU activation function, and $W_{d}^{\left(m\right)}$ and $b_{d}^{\left(m\right)}$ are the weight matrix and bias parameters, respectively. The output of the decoder's final layer yields the reconstructed data ${\tilde{X}}^{t}$. All parameters of the AE model are optimized using the following loss function:

(3) $$L_{ae}=\frac{1}{c^{t}}\sum_{i=1}^{c^{t}}\sum_{j=1}^{p}{\left(X_{ij}^{t}-{\tilde{X}}_{ij}^{t}\right)}^{2}.$$

### Graph Construction

After training the autoencoder (AE) model, we project the reference‐target data into a common latent space using the trained AE to obtain their low‐dimensional representations. Based on these latent representations, we employ the cosine similarity algorithm to identify the most similar samples for each sample within and between the reference and target data. Using these similarity scores, we construct three graphs: the reference graph within the reference data, the target graph within the target data, and the joint graph between the reference and target data. Each graph is represented as an adjacency matrix.

For instance, to establish sample‐sample relationships within the reference data, we first extract the low‐dimensional representations of all samples from the trained AE, denoted as $Z^{r}\in R^{N_{r}\times d}$, where *N*_*r*_ is the number of samples and *d* is the latent dimension size. The similarity between samples *s*_*i*_ and *s*_*j*_ in the reference data is computed using cosine similarity as follows:

(4) $$s_{\left(ij\right)}^{r}=\frac{Z_{:,i}^{r}\cdot Z_{:,j}^{r}}{∥Z_{:,i}^{r}{∥}_{2}{∥Z_{:,j}^{r}∥}_{2}},$$

where $Z_{:,i}^{r}$ and $Z_{:,j}^{r}$ represent the *i*‐th and *j*‐th columns of the matrix *Z* ^*r*^. The similarity matrix $S_{r}\in R^{N_{r}\times N_{r}}$ captures all pairwise sample relationships within the reference data. Similarly, we compute the similarity matrix for the target data, denoted as $S_{t}\in R^{N_{t}\times N_{t}}$. For the joint relationships between the reference and target data, we concatenate their low‐dimensional representations into a combined matrix $Z^{r+t}\in R^{N_{r+t}\times d}$ and construct the joint similarity matrix $S_{r+t}\in R^{N_{r+t}\times N_{r+t}}$. These matrices are used for information propagation through a graph neural network, where samples are represented as nodes and similarity relationships define the adjacency matrix.

### Graph Construction—Re‐weighting Edges

Since the cell type composition of each sample in the reference data is known, we re‐weight the similarity scores in the reference similarity matrix based on the ground truth cell type composition, assigning larger weights to edges with stronger homophily. This enhances the representational ability of the GNN and improves performance, as demonstrated in previous studies.^[^ ^25^ ^]^ Specifically, we quantify the homophily of each edge using ground truth or predicted label distributions and assign larger weights to edges with higher similarity. This module is heuristic and requires no training. Concretely, we measure the homophily of edge (*i*, *j*) using the cosine similarity between the label predictions $z_{i}^{label}$ and $z_{j}^{label}$, and define the edge weight as:

(5) $$w_{i,j}=\frac{z_{i}^{label}\cdot z_{j}^{label}}{∥z_{i}^{label}{∥}_{2}{∥z_{j}^{label}∥}_{2}}.$$

Here, *w*_*i*, *j*_ is used to improve the calibration performance of the GNN. In this study, we assign an edge weight of $1.5\times s_{\left(ij\right)}^{r}$ if *w*_*i*, *j*_ > 0.5; otherwise, the edge weight is set to $0.8\times s_{\left(ij\right)}^{r}$.

### The Graph Neural Network

Building on the graph construction described above section, we now introduce a graph neural network (GNN) framework designed to learn expressive sample representations. The model operates on three types of graphs: a reference graph capturing known cell composition patterns, a target graph modeling internal structure in the target dataset, and a joint graph facilitating information transfer between the reference and target domains. Protein samples are treated as nodes in these graphs, and their pairwise similarities define the adjacency structure.

The adjacency matrices used in the GNN are derived from the similarity matrices introduced in the Graph Construction section. These similarity matrices define undirected, weighted graphs that support neighborhood‐based feature propagation. We denote the overall graph structure as follows:

(6) $$G=\left(V,E\right),$$

where *V* = *V*_*r*_∪*V*_*t*_ is the set of nodes, comprising nodes from the reference dataset *V*_*r*_ and the target dataset *V*_*t*_. The edge set is defined as:

(7) $$E=E_{r}\cup E_{t}\cup E_{j},$$

where *E*_*r*_ represents the edges within the reference dataset (constructed from similarity matrix *S*_*r*_), *E*_*t*_ denotes the edges within the target dataset (from *S*_*t*_), and *E*_*j*_ represents the edges across reference and target samples, derived from the joint similarity matrix *S*_*r* + *t*_.

Although we present the edge sets together for notational convenience, the GNN model processes each graph independently during training. Specifically, in each epoch: The reference graph *G*_*r*_ = (*V*_*r*_, *E*_*r*_) and the target graph *G*_*t*_ = (*V*_*t*_, *E*_*t*_) are separately passed through the GNN to obtain their respective node representations. In parallel, the joint graph *G*_*j*_ = (*V*_*r*_∪*V*_*t*_, *E*_*j*_) is processed to extract shared representations that capture global relational patterns across datasets.

To perform feature propagation, we adopt GraphSAGE as the backbone of our message‐passing mechanism. At each convolutional layer *k*, the hidden representation of a node *v* is updated by aggregating information from its neighborhood:

(8) $$h_{N(v)}^{\left(k\right)}=\frac{1}{|N_{k}\left(v\right)|}\sum_{u\in N_{k}\left(v\right)}h_{u}^{\left(k-1\right)},$$

where *N*_*k*_(*v*) denotes the set of neighbors of node *v* at layer *k*, and $h_{u}^{\left(k-1\right)}$ is the representation of node *u* from the previous layer. The updated embedding of node *v* is then computed by concatenating its previous representation with the aggregated neighborhood embedding:

(9) $$h_{v}^{\left(k\right)}=\sigma \left(W_{k}\cdot \text{concat}\left(h_{v}^{\left(k-1\right)},h_{N(v)}^{\left(k\right)}\right)\right),$$

where *W*_*k*_ is a trainable weight matrix and σ is a non‐linear activation function (e.g., ReLU). This process is repeated for *L* layers to capture multi‐hop neighborhood information.

To integrate representations across layers, we perform a layer‐wise weighted summation to generate the final multi‐hop node representation:

(10) $$M_{v}=\sum_{l=1}^{L}s_{v}^{l}\times h_{v}^{l},$$

where $s_{v}^{l}$ denotes the learned importance weight of layer *l* for node *v*, with $\sum_{l=1}^{L}s_{v}^{l}=1$. In our experiments, we set *L* = 2.

To capture diverse structural and semantic patterns, we employ a multi‐channel architecture where each channel represents an independent GraphSAGE model initialized differently. Each channel processes the same graph structure (reference, target, or joint), but learns to encode complementary perspectives. For each node *v*, its final representation is constructed by concatenating the outputs from all *C* channels:

(11) $$Z_{GNN}=\left[M_{v}^{1},M_{v}^{2},\text{…},M_{v}^{C}\right],$$

where $M_{v}^{c}$ is the final representation of node *v* from channel *c*. The resulting node embeddings are then passed to a multi‐layer perceptron (MLP) module and optimized jointly using multiple task‐specific losses.

### The Loss Functions

To optimize the GNN model, we employ three loss functions: (1) a triplet loss function to attract similar samples and repel dissimilar ones, (2) a Mean Squared Error (MSE) loss function to recover cell type composition, and (3) a domain adaptation loss function to distinguish between reference and target data.

### The Loss Functions—Prediction MSE Loss

To predict cell‐type proportions from the learned representations, we first process the input data using a Graph Neural Network (GNN). The learned node representation $z_{GNN}^{i}$ from the GNN module encodes both local and global features of the spot, incorporating information from its neighboring nodes in the graph. After the GNN processes the graph, we employ a deconvolution head, implemented as a multi‐layer perceptron (MLP), to map these learned node representations to predicted cell‐type proportions for each spot. Specifically, for each spot *i*, the deconvolution head produces a cell‐type proportion vector *p*_*i*_ as follows:

(12) $$p_{i}=\mathrm{Softmax}\left(\mathrm{MLP}\left(z_{GNN}^{i}\right)\right),$$

where *p*_*i*_ is the predicted cell‐type proportion vector for spot *i*, and the softmax function ensures that the predicted proportions sum to one, providing a valid probability distribution over cell types. The MLP used in the deconvolution head consists of fully connected layers with nonlinear activation functions (e.g., ReLU), allowing it to learn complex mappings from the latent space of spot representations to cell‐type proportions.

We then compute the mean squared error (MSE) between the predicted cell‐type abundances *p*_*i*_ and the corresponding ground truth abundance labels *Y* ^*r*^ for the reference data. The MSE loss is defined as follows:

(13) $$L_{dec}=\left|\right|Y^{r}-p_{i}{\left|\right|}_{2}^{2}$$

where *Y* ^*r*^ is the true ground truth abundance label for the reference dataset. The loss function *L*_*dec*_ is used to optimize the model weights by minimizing the discrepancy between the predicted and true cell‐type abundance values. By minimizing this loss, the model learns to accurately map the learned representations from the GNN to meaningful estimates of cell‐type proportions, improving the overall accuracy of cell‐type deconvolution in our model.

### The Loss Functions—Triplet Loss

To further attract similar samples and repel dissimilar ones, we employ the triplet loss. Specifically, for each anchor sample in the reference and target data, we select one positive sample and one negative sample based on the similarity matrices constructed in the graph construction section. For each sample in the similarity matrix, the sample with the highest similarity is chosen as the positive sample, while the sample with the lowest similarity is selected as the negative sample. Subsequently, we aim to bring the latent encoding of the anchor sample closer to that of the positive sample and push it farther from that of the negative sample. The triplet loss function is formulated as follows:

(14) $$\begin{array}{c} L_{tri}=\sum_{i=1}^{r+t}\max\left(\frac{z_{i,:}^{r+t}\cdot z_{neg_{i},:}^{r+t}}{|z_{i,:}^{r+t}\left|\right|z_{neg_{i},:}^{r+t}|}-\frac{z_{i,:}^{r+t}\cdot z_{pos_{i},:}^{r+t}}{|z_{i,:}^{r+t}\left|\right|z_{pos_{i},:}^{r+t}|}+m,0\right)/\left(r+t\right) \end{array}$$

where *m* is a non‐negative hyperparameter, primarily used to prevent the model from taking shortcuts, ensuring that the latent encodings of negative and positive samples are not trained to be too close.

### The Loss Functions—Domain adaptation loss

The domain adaptation loss is employed to mitigate batch effects between the reference and target data. We iteratively train the model using reference/target domain labels to ensure that it extracts domain‐invariant features for the deconvolution task across different sources. Specifically, for the latent encodings $z_{GNN}^{r}$ and $z_{GNN}^{t}$ of the reference and target data, we feed them into a domain discriminator, implemented as a two‐layer perceptron, to predict the domain to which the data belongs. We utilize a binary cross‐entropy loss function to quantify the prediction errors, as defined by the following formula:

(15) $$\begin{array}{c} d^{r}=\mathrm{Dom}\left(z_{GNN}^{r}\right) \end{array}$$

(16) $$\begin{array}{c} d^{t}=\mathrm{Dom}\left(z_{GNN}^{t}\right) \end{array}$$

(17) $$\begin{array}{c} L_{dom}=\log\left(1-d_{GNN}^{t}\right)+\log\left(d_{GNN}^{r}\right) \end{array}$$

where Dom(·) represents the domain discriminator, implemented as a multi‐layer perceptron (MLP).

The final training loss is a weighted sum of the prediction loss, triplet loss, and domain adaptation loss, given by:

(18) $$L=L_{dec}+\alpha \cdot L_{tri}+\beta \cdot L_{dom}$$

where α and β are the contribution weights of the triplet loss and the domain adaptation loss, respectively.

### Implementation Details

GraphDEC is implemented using PyTorch and Python. The autoencoder architecture is designed with dimensions [*input*‐16‐*input*], optimized using the Adam optimizer with a learning rate of 0.001. For the Graph Neural Network (GNN), the latent hidden dimension is set to 256, also optimized with the Adam optimizer at a learning rate of 0.001. The hyperparameter *m* for the triplet loss is set to 0.3, with the loss function weights configured as α = 0.01 and β = 1. During the training phase, the model was trained for 3, 000 iterations, with 10% of the training set reserved for validation. Training continued for at least 1, 500 iterations, with early stopping triggered if the validation metric did not improve for 100 consecutive iterations. Each epoch consisted of two steps: First, the reference and target data, along with the reference graph and target graph, were fed into GraphDEC, and three loss functions were computed, including the triplet loss, mean squared error (MSE) loss based on training set labels, and domain adaptation loss (using true domain labels). Second, the reference and target data, along with the joint graph, were passed through the model, and only the triplet loss and training set prediction loss were computed. In the prediction phase, the target data with the target graph were input into the model, and the prediction head generated an initial estimate of cell abundance. These initial prediction labels were used to re‐weight the target graph. The adjusted target graph was then passed through the model again to obtain the final cell abundance prediction. The experiments described in this paper were conducted on a system running Ubuntu 18.04.7 LTS, equipped with an Intel^®^ Core™ i7‐8700K CPU @ 3.70 GHz, 256 GB RAM, and an NVIDIA GeForce RTX 4090 GPU.

### Evaluation Metrics

Following the methodologies of *Scaden* and *scpDeconv*, we employed three widely recognized metrics to assess the performance of our approach: Lin's concordance correlation coefficient (CCC), root mean square error (RMSE), and Pearson correlation coefficient (PCC). These metrics were chosen to provide a comprehensive evaluation of both the accuracy and consistency of the predicted cell type proportions against the ground truth.

### Evaluation Metrics—Lin's Concordance Correlation Coefficient (CCC)

 measures the agreement between predicted and true values while accounting for both precision and accuracy. CCC is defined as:

(19) $$\mathrm{CCC}\left(y,\hat{y}\right)=\frac{2\cdot \mathrm{PCC}\left(y,\hat{y}\right)\cdot \sigma_{y}\sigma_{\hat{y}}}{\sigma_{y}^{2}+\sigma_{\hat{y}}^{2}+{\left(\mu_{y}-\mu_{\hat{y}}\right)}^{2}}$$

where *y* and $\hat{y}$ represent the true and predicted cell type abundance proportions, respectively, σ_*y*_ and $\sigma_{\hat{y}}$ denote their standard deviations, µ_*y*_ and $\mu_{\hat{y}}$ are their means, and $\mathrm{PCC}(y,\hat{y})$ is the Pearson correlation coefficient.

### Evaluation Metrics—Root Mean Square Error (RMSE)

 quantifies the average magnitude of prediction errors and is defined as:

(20) $$\mathrm{RMSE}\left(y,\hat{y}\right)=\sqrt{\mathrm{avg}\left({\left(y-\hat{y}\right)}^{2}\right)}$$

A lower RMSE indicates better agreement between predictions and the ground truth.

**Pearson Correlation Coefficient (PCC)** measures the linear correlation between the predicted and true cell type proportions, capturing the strength and direction of the relationship:

(21) $$\mathrm{PCC}\left(y,\hat{y}\right)=\frac{\mathrm{cov}(y,\hat{y})}{\sigma_{y}\sigma_{\hat{y}}}$$

where $\mathrm{cov}(y,\hat{y})$ represents the covariance between *y* and $\hat{y}$. A PCC close to 1 suggests a strong positive correlation.

### Data Availability

All datasets utilized in this study are publicly accessible via their respective GEO accession numbers or direct URLs. The detailed information is provided below:

**The Murine_cellline dataset**, derived from mouse and sequenced using single‐cell proteomics technologies N2^[^ ^30^ ^]^ and nanoPOTS,^[^ ^31^ ^]^ includes three mouse cell lines: RAW 264.7 (macrophage cell line), C10 (respiratory epithelial cell line), and SVEC (endothelial cell line), with a shared set of 762 proteins. The dataset is publicly available at https://github.com/TencentAILabHealthcare/scpDeconv/tree/main/data/murine_cellline. The N2‐sequenced data was used to construct the reference dataset, while the nanoPOTS‐sequenced data served as the target dataset. We directly utilized 1,000 pseudo‐samples for the target dataset and 4,000 pseudo‐samples for the reference dataset, generated using the methodology described in [22], to evaluate each method.

**The HBreast_CYTOF dataset** ^[^ ^32^ ^]^ comprises 38 human breast tissue samples, encompassing six common cell types (alveolar cells, hormone‐sensing cells, basal cells, fibroblasts, vascular lymphatic cells, and immune cells) and 34 protein markers. The data was generated using CyTOF technology and obtained from https://data.mendeley.com/datasets/vs8m5gkyfn/1. For our analysis, batch 0 of the HBreast_CYTOF dataset was used to construct the reference data, while batch 1 served as the target data. Pseudo‐samples were generated by randomly selecting 15, 50, or 200 single‐cell data points from the single‐cell proteomics data and fusing them into a single pseudo‐sample. This process was repeated to create 20,000 pseudo‐samples for the reference dataset and 4,000 pseudo‐samples for the target dataset.

**The PBMC_REAP dataset** comprises two batches of human single‐cell data, each containing thousands of cells with 35 common proteins and 12 cell types (B_cell, CMP, DC, GMP, HSC_‐G‐CSF, MEP, Monocyte, NK_cell, Platelets, Pre‐B_cell_CD34‐, Pro‐B_cell_CD34+, T_cells). The data was generated using REAP‐seq technology and is available through GEO accession numbers GSM2685243 and GSM2685244. GSM2685243 was used to construct the reference dataset, while GSM2685244 served as the target dataset. Pseudo‐samples were generated by randomly selecting 15, 50, or 200 single‐cell data points and fusing them into a single pseudo‐sample. This process was repeated to create 20,000 pseudo‐samples for the reference dataset and 4,000 pseudo‐samples for the target dataset.

**The BMMC dataset**, derived from bone marrow mononuclear cells of 12 healthy human donors,^[^ ^33^ ^]^ comprises 36 sub‐cell types and 14,087 protein markers. The dataset was generated using CITE‐seq technology and is available through GEO accession number GSE194122. For our analysis, batch s1d1 was used to construct the reference dataset, while batch s1d2 served as the target dataset. Pseudo‐samples were generated by randomly selecting 15, 50, or 200 single‐cell data points and fusing them into a single pseudo‐sample. This process was repeated to create 4,000 pseudo‐samples for the reference dataset and 1,000 pseudo‐samples for the target dataset.

**The PBMC_CITE dataset**, derived from human samples and sequenced using CITE‐seq technology, comprises 8 cell types and 2,28 proteins. The data was obtained from ref. [34] and is available at https://github.com/single‐cell‐proteomic/SCPRO‐HI/tree/main/Data/PBMC. For our analysis, batch 1 was used to construct the reference dataset, while batch 2 served as the target dataset. Pseudo‐samples were generated by randomly selecting 15, 50, or 200 single‐cell data points and fusing them into a single pseudo‐sample. This process was repeated to create 20,000 pseudo‐samples for the reference dataset and 4,000 pseudo‐samples for the target dataset.

**The four cross‐species datasets—H1N1Human, IFHuman, IFrhesus, and IFcynomolgus**—were obtained from the studies^[^ ^35^, ^36^ ^]^ and are publicly available at https://github.com/single‐cell‐proteomic/SCPRO‐HI/tree/main/Data/cross‐species. These datasets were derived from 35 healthy humans challenged with the H1N1 virus (H1N1Human), 86 humans stimulated with IFNγ (IFHuman), 32 rhesus macaques stimulated with IFNγ (IFrhesus), and 32 cynomolgus monkeys stimulated with IFNγ (IFcynomolgus). The H1N1Human dataset was measured by mass cytometry, while the others were measured by phospho‐flow immune signaling. Each dataset contains 120,000 blood cells categorized into seven cell types. Pseudo‐samples for each reference‐target dataset were generated by randomly selecting 15, 50, or 200 single‐cell data points and fusing them into a single pseudo‐sample. This process was repeated to create 20,000 pseudo‐samples for the reference dataset and 4,000 pseudo‐samples for the target dataset.

**The Human palatine tonsil dataset** includes reference data and spatial proteomics data. The reference data, obtained from Zenodo (https://zenodo.org/records/8373756), contains processed CITE‐seq data with 192 proteins across 42,929 cells from 15 donors. To reduce biases from patient or batch effects, we used the donor “BCLL‐15‐T” (17,371 cells from batch “BCLLATLAS_46”) as the reference. The spatial data, downloaded from Gene Expression Omnibus (GSE213264), includes 282 proteins for 2,492 spots. Manual curation identified 174 proteins common to both datasets. For the reference dataset, 20,000 pseudo‐samples were generated by randomly selecting and fusing 50 single‐cell data points into each pseudo‐sample.

**The Mouse PDAC dataset** includes reference data and spatial proteomics data. The reference data, obtained from Zenodo (https://doi.org/10.5281/zenodo.13978420), contains 5,900 proteins across 14 mouse PDAC cell types. We selected 10 primary cell types, each from 5 KP f/f C mice, and retained 5,788 proteins from 50 cells after filtering for at least two valid values per cell type. The spatial data, available at Zenodo (https://zenodo.org/records/14233847), includes 3,607 proteins from 108 spots. A total of 2,837 proteins were common to both datasets. For the reference dataset, 1,000 pseudo‐samples were generated by randomly selecting and fusing 15 single‐cell data points into each pseudo‐sample.

**All transcriptomics datasets—Mousebrain, Pancreas, and PBMC_data**—were obtained from the Scaden study^[^ ^6^ ^]^ and are available at https://scaden.readthedocs.io/en/latest/datasets.html. For these datasets, we directly utilized the preprocessed data provided by Scaden, which included pre‐constructed reference‐target pairs and standardized data.

## Conflict of Interest

The authors declare no conflict of interest.

## Author Contributions

Z.D. and Y.S. contributed equally to this work. Y.Z. and Z.D. conceived and supervised the project. Y.Z. and Y.S. contributed to the algorithm implementation. Y.Z. and Z.D. wrote the manuscript. Y.S., H.Z., H.Y.Z., Z.W., T.Q., and Y.Y. were involved in the discussion and proofreading.

## Supporting information

Supporting Information

## Data Availability

All datasets are described in detail in the Data Availability section of the manuscript.
