# Supplementary material for: Deciphering Cell Type Abundance in Proteomics Data Through Graph Neural Networks
Source: Adv Sci (Weinh). 2025 Jun 20;12(33):e02987. doi: 10.1002/advs.202502987 (PMC12412507; doi:10.1002/advs.202502987)
Supplement: Supplementary file 1 — Supporting Information [file ADVS-12-e02987-s001.pdf]

ADVANCED SCIENCE
Open Access

Supporting Information

for *Adv. Sci.*, DOI 10.1002/advs.202502987

Deciphering Cell Type Abundance in Proteomics Data Through Graph Neural Networks

*Zhiming Dai, Yujie Song, Tuoshi Qi, Hongyu Zhang, Huiying Zhao, Zheng Wang, Yuedong Yang and Yuansong Zeng**

Supplementary Materials for Deciphering Cell Type Abundance in Proteomics Data through Graph Neural Networks

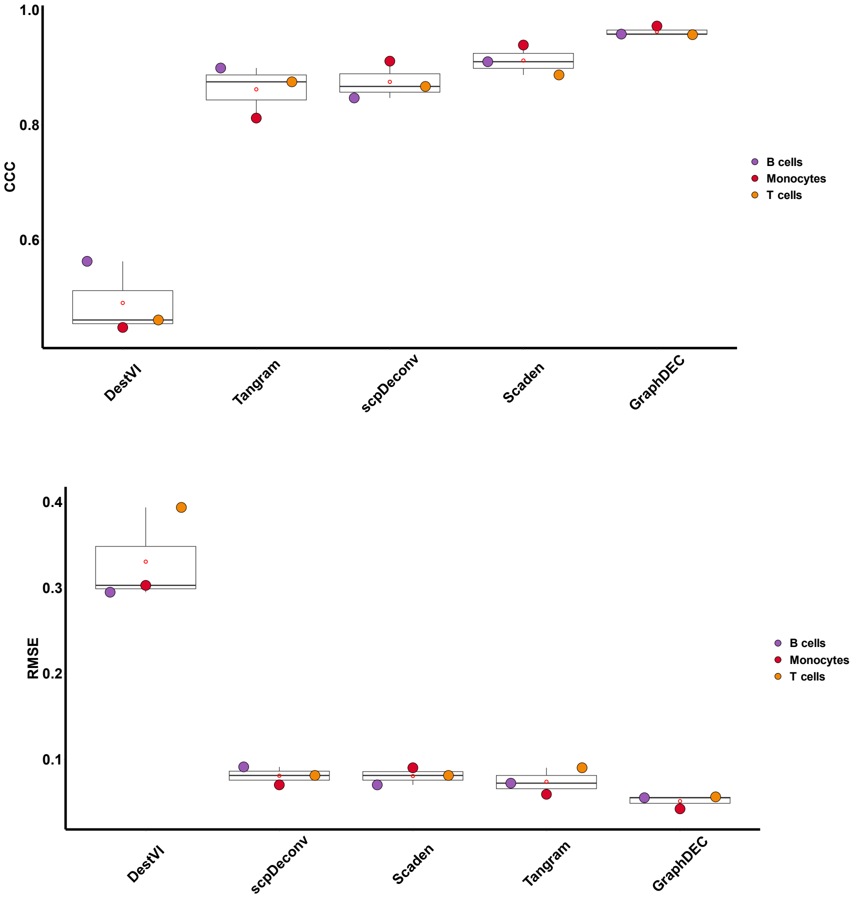

Fig. S1 Boxplots of CCC and RMSE values for each deconvolution method on the PBMC_REAP. The boxplots are defined as follows: the minimum is the 25th percentile minus 1.5 times the interquartile range (IQR), and the maximum is the 75th percentile plus 1.5 times the IQR. The box hinges represent the IQR, while the whiskers extend to 1.5 times the IQR. The center line indicates the median, and the box bounds correspond to the 25th and 75th percentiles. An open red circle denotes the mean value, and black dots represent outliers. This analysis is based on n = 3 cell types. The x-axis labels the deconvolution methods, and the y-axis represents the CCC and RMSE values, respectively.

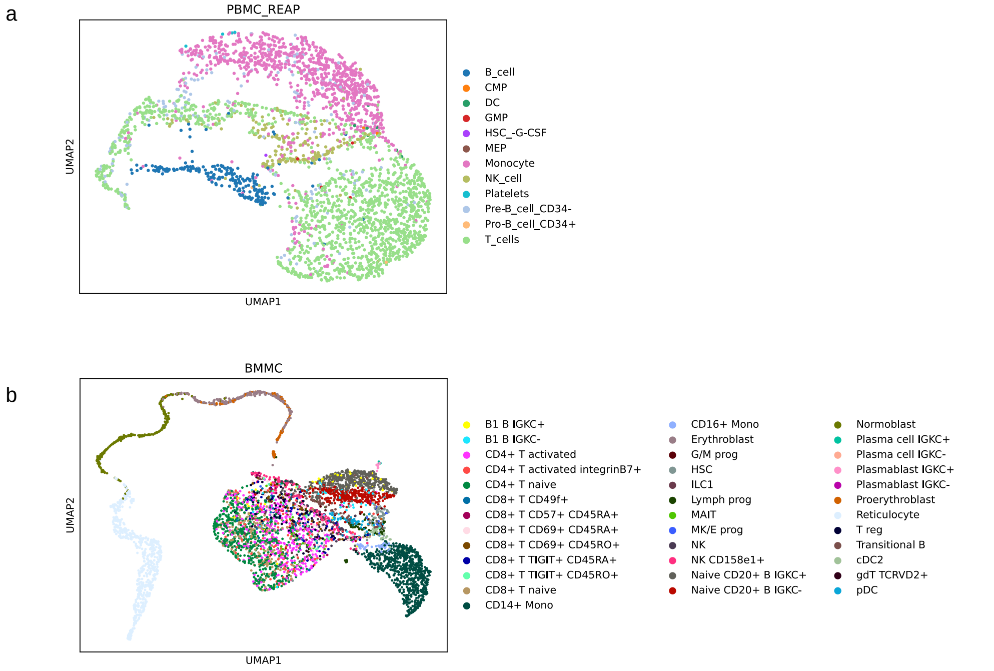

Fig. S2 UMAP scatter plots of the single-cell (a) PBMC_REAP and (b) BMMC proteomics data, which are colored by cell types.

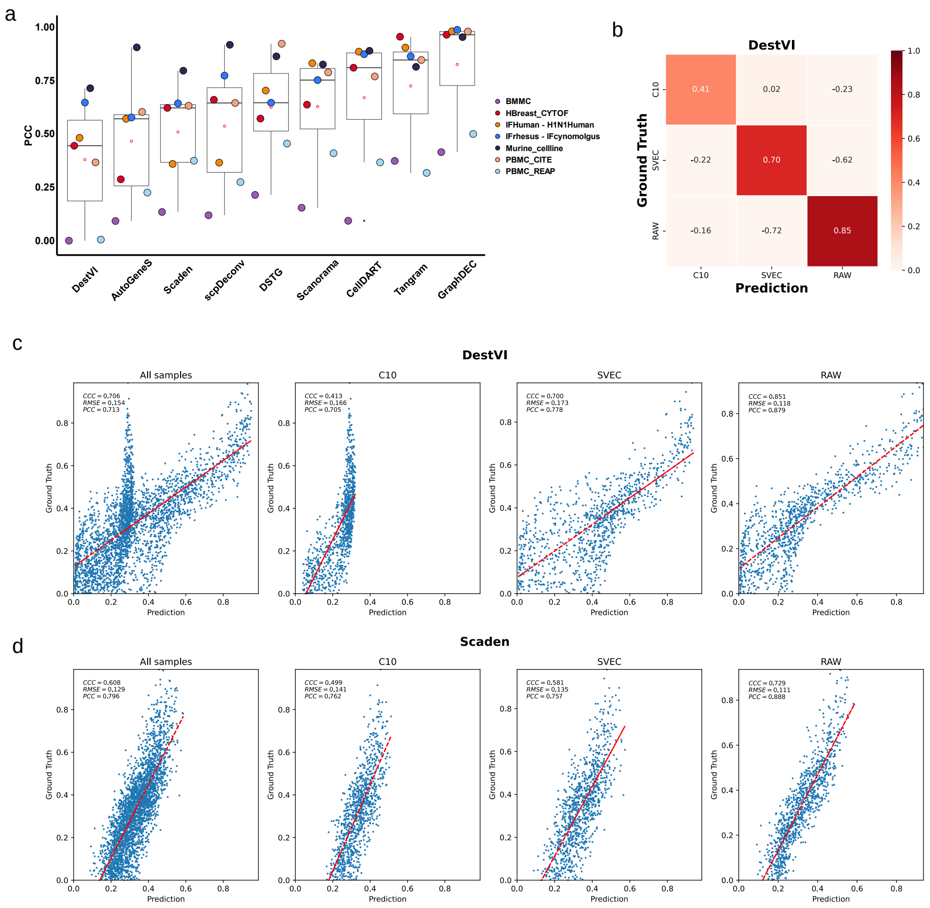

Fig. S3 (a) Boxplots showed PCC values for each method. The boxplots are defined as follows: the minimum is calculated as the 25th percentile minus 1.5 times the interquartile range (IQR), and the maximum is calculated as the 75th percentile plus 1.5 times the IQR. The hinges of the box represent the interquartile range (IQR), while the whiskers extend to 1.5 times the IQR. The center line of the boxplot indicates the median and the bounds of the box correspond to the 25th and 75th percentiles. An open red circle denotes the average values within each boxplot, and black dots represent outliers. This analysis is based on n = 7 biologically independent reference-target proteomics datasets. The x-axis labels the different deconvolution methods, and the y-axis represents the measured values. (b) The confusion matrices of the prediction results from method DestVI on the Murine_cellline dataset. The diagonal cells represent the CCC values between the predicted and true values for each specific cell type. (c) and (d) Scatter plots of the ground truth (y-axis) and predicted (x-axis) proportions of three cell types (C10, SVEC, and RAW) in the case of Murine cell line datasets using DestVI and Scaden, respectively.

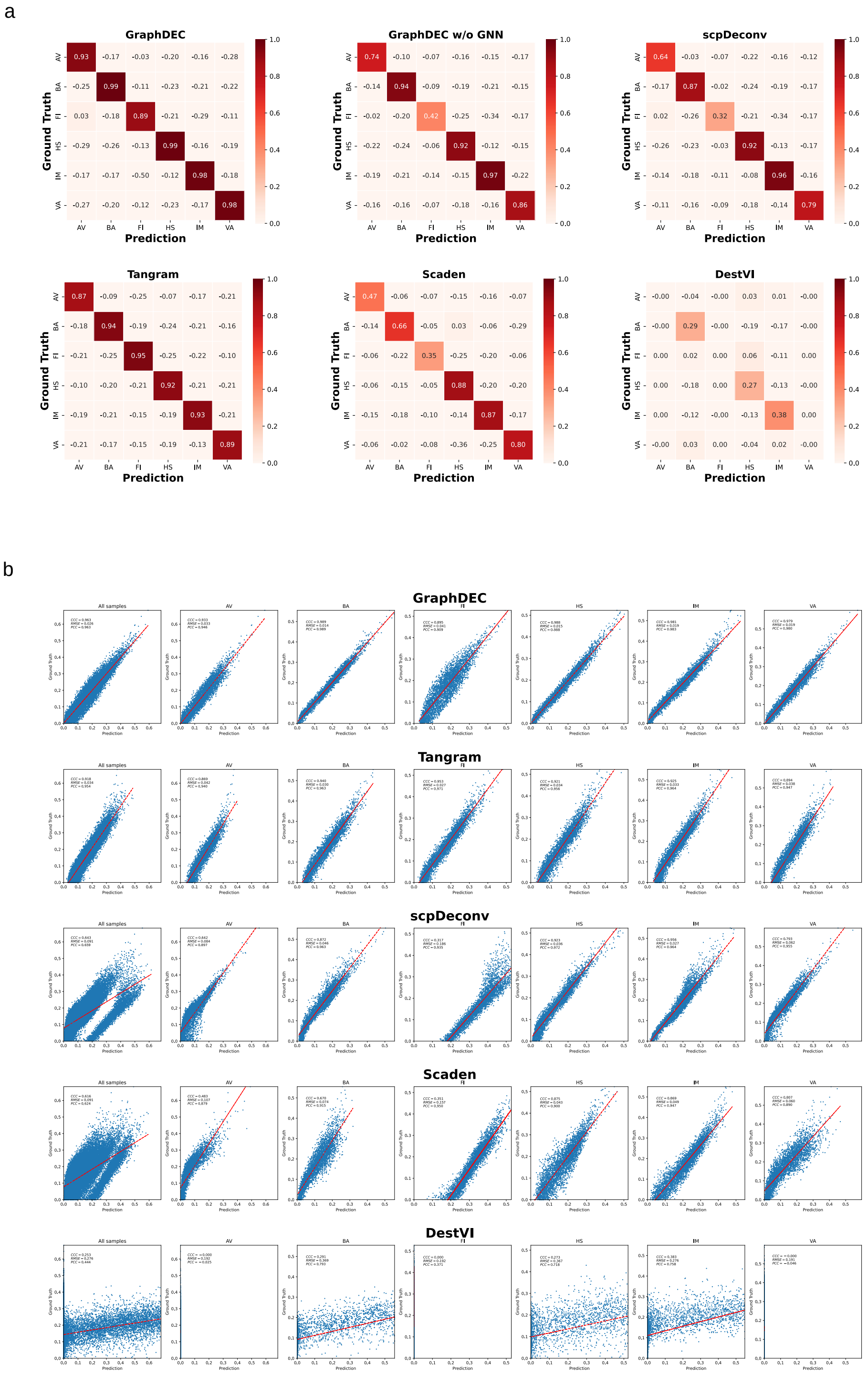

Fig. S4 (a) The confusion matrices of the prediction results from competing methods, GraphDEC without GNN, and GraphDEC on the HBreast_CYTOF dataset. The diagonal cells represent the CCC values between the predicted and true values for each specific cell type. (b) Scatter plots of the ground truth (y-axis) and predicted (x-axis) proportions of three cell types (AV, BA, FI, HS, IM, and VA) in the case of HBreast_CYTOF datasets using each method.

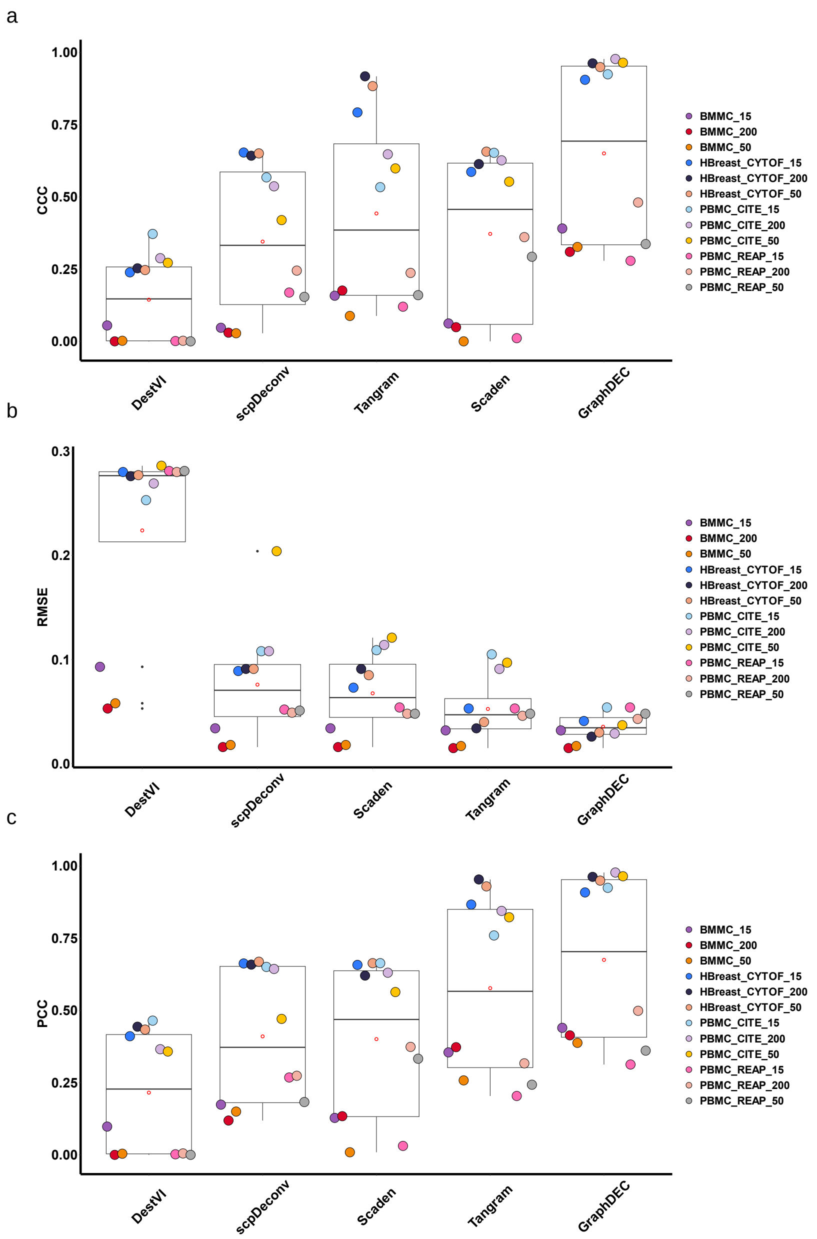

Fig. S5 The performance of each method when changing the number of mixed cells in each sample of the reference data.

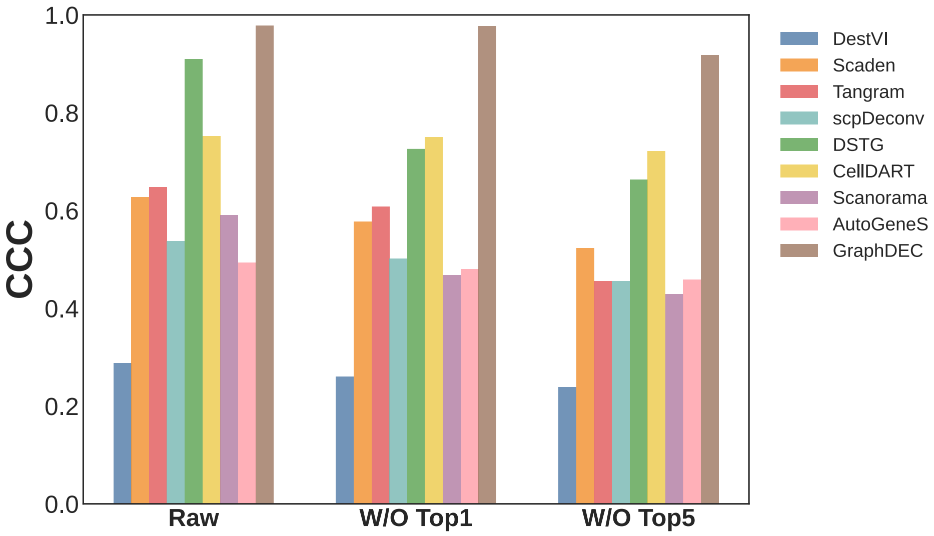

Fig. S6 Evaluation of deconvolution methods on raw proteomic data and on modified datasets where the top 1 marker protein or the top 5 differentially expressed proteins were removed for each cell type.

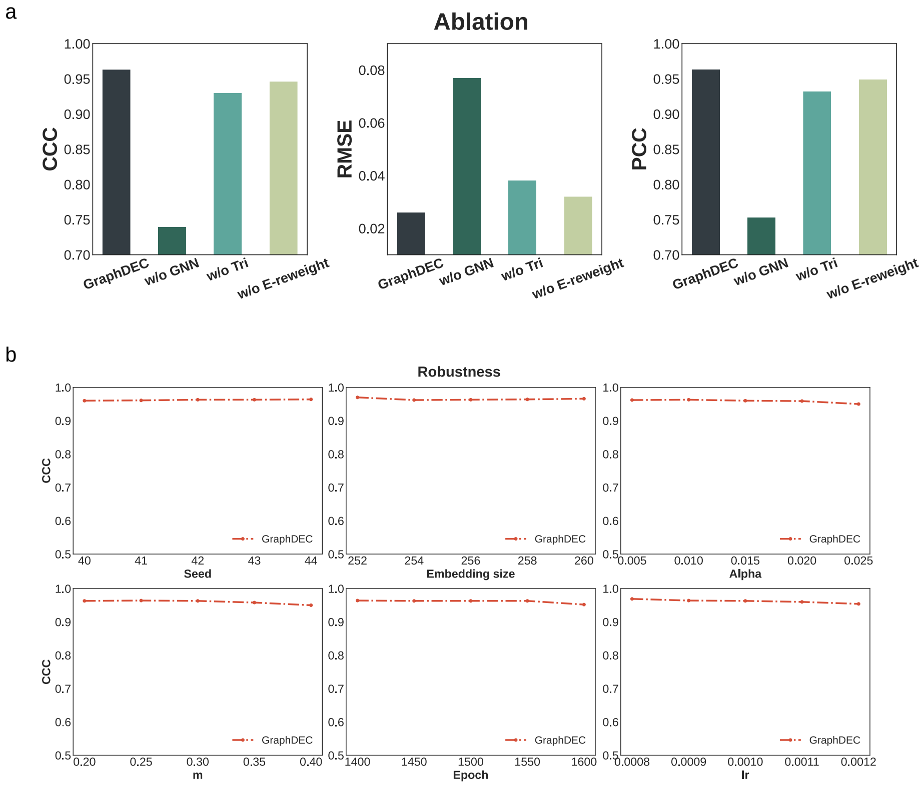

Fig. S7 (a) Ablation study of GraphDEC showing the impact of removing the Graph Neural Network (GNN) module, the triplet loss (Tri), and the edge weight reweighting mechanism (E-reweight). Performance is evaluated using CCC, RMSE, and PCC metrics. (b) Sensitivity analysis of GraphDEC performance with respect to key hyperparameter variations.

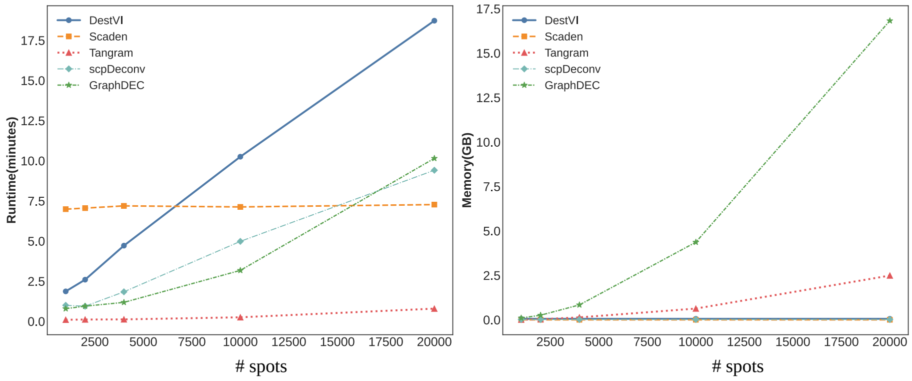

Fig. S8 Training time and memory consumption of each deconvolution method, evaluated under consistent experimental settings.

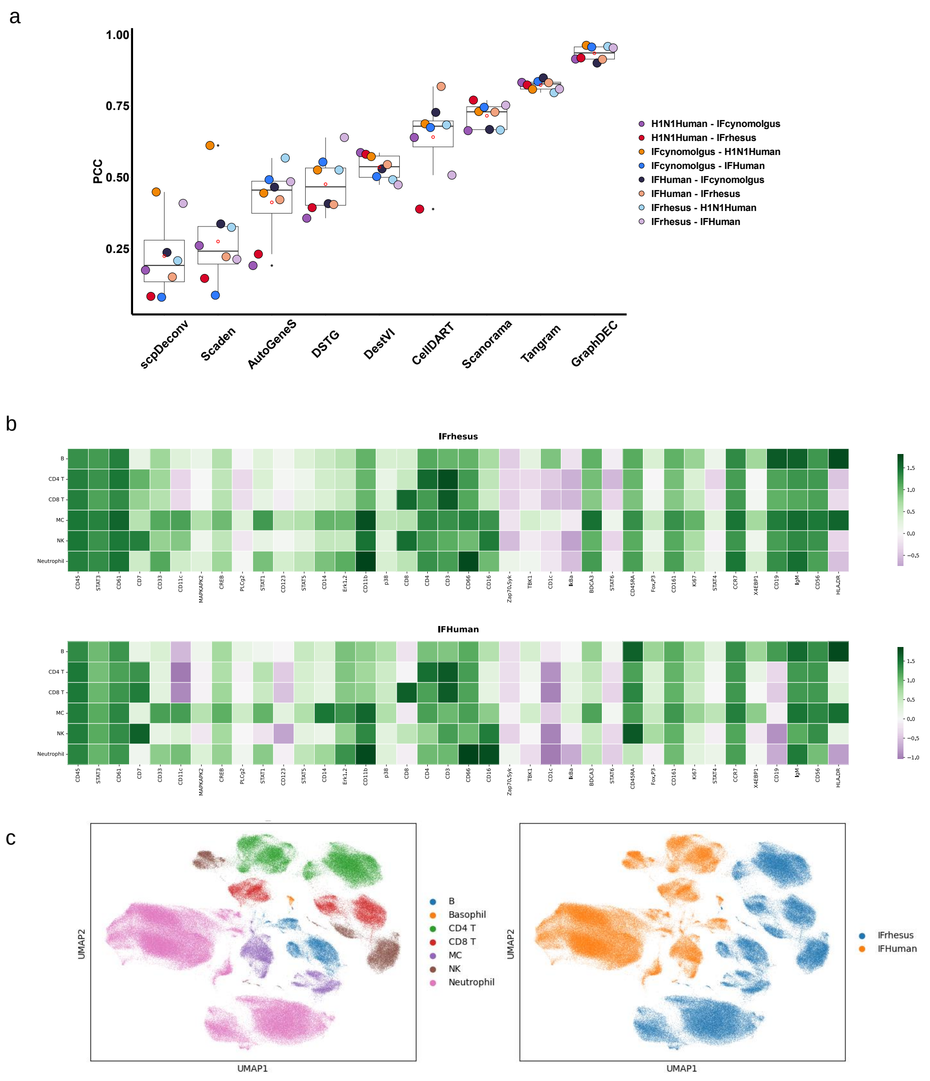

Fig. S9 (a) Boxplots showed PCC values for each method. The boxplots are defined as follows: the minimum is calculated as the 25th percentile minus 1.5 times the interquartile range (IQR), and the maximum is calculated as the 75th percentile plus 1.5 times the IQR. The hinges of the box represent the interquartile range (IQR), while the whiskers extend to 1.5 times the IQR. The center line of the boxplot indicates the median and the bounds of the box correspond to the 25th and 75th percentiles. An open red circle denotes the average values within each boxplot, and black dots represent outliers. This analysis is based on n = 8 biologically independent reference-target cross-species datasets. The x-axis labels the different deconvolution methods, and the y-axis represents the measured values. (b) Heatmap of the abundance of key proteins in IFrhesus and IFHuman datasets, respectively. (c) UMAP scatter plot of the single-cell cross-species proteomics data used for constructing the reference data of the IFrhesus-IFHuman data, which are colored by cell types and species.

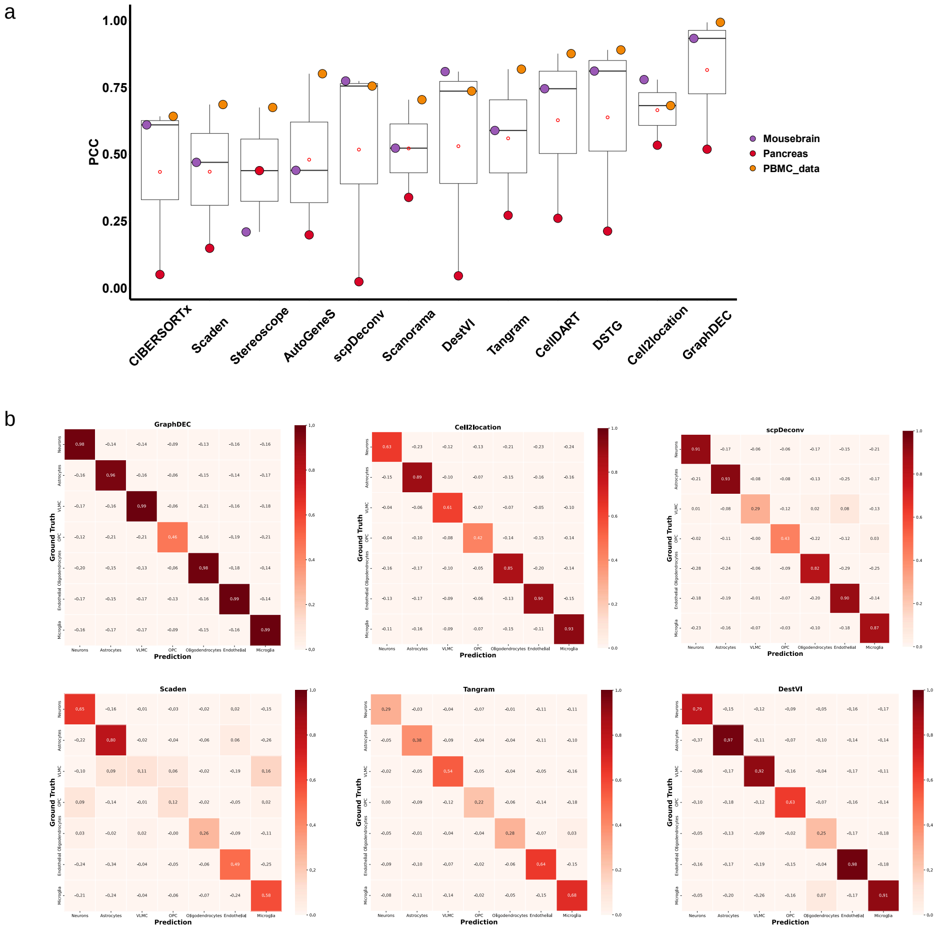

Fig. S10 (a) Boxplots showed PCC values for each method. The boxplots are defined as follows: the minimum is calculated as the 25th percentile minus 1.5 times the interquartile range (IQR), and the maximum is calculated as the 75th percentile plus 1.5 times the IQR. The hinges of the box represent the interquartile range (IQR), while the whiskers extend to 1.5 times the IQR. The center line of the boxplot indicates the median and the bounds of the box correspond to the 25th and 75th percentiles. An open red circle denotes the average values within each boxplot, and black dots represent outliers. This analysis is based on n = 3 biologically independent reference-target transcriptomics datasets. The x-axis labels the different deconvolution methods, and the y-axis represents the measured values. (b) The confusion matrices of the prediction results from each method on the Mousebrain dataset. The diagonal cells represent the CCC values between the predicted and true values for each specific cell type.

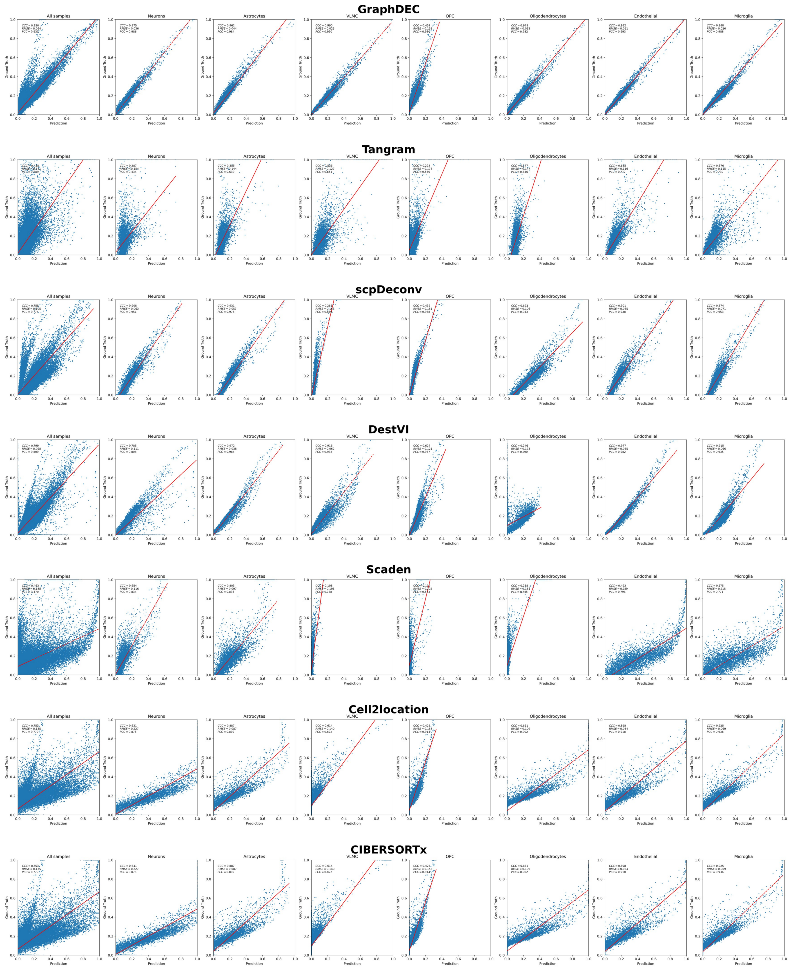

Fig. S11 Scatter plots of the ground truth (y-axis) and predicted (x-axis) proportions of three cell types (Neurons, Astrocytes, VLMC, OPC, Oligodendrocytes, Endothelial, and Microglia) in the case of Mousebrain data using each method.

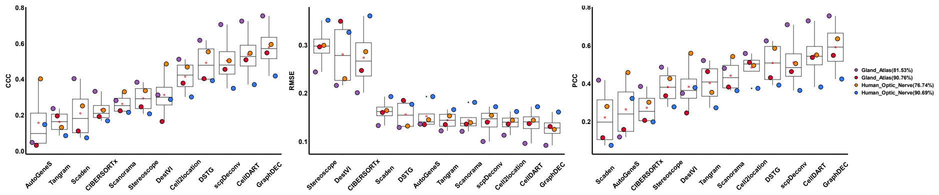

Fig. S12 Performance comparison of different methods on dropout-heavy transcriptomics datasets, including Human_Optic_Nerve and Gland_Atlas, evaluated using CCC, RMSE, and PCC. In the legend, 81.53% indicates the dropout rate of the original Gland_Atlas dataset, while 90.76% corresponds to the dropout rate of its downsampled version. The same applies to the Human_Optic_Nerve dataset, with dropout rates of 76.74% (original) and 90.69% (downsampled), respectively.

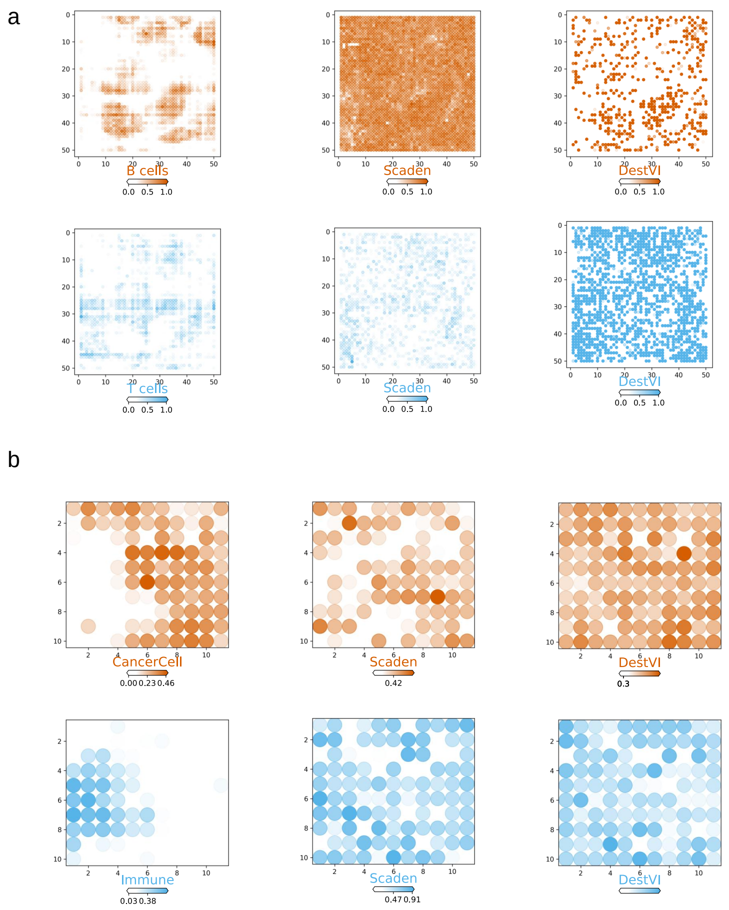

Fig. S13 (a) Heatmaps showing the spatial abundance of B cells and T cells predicted by Scaden and DestVI, respectively, in the human_palatine_tonsil dataset. The x and y axes represent the true spatial locations in the section as provided by the target Tonsil dataset. (b) Heatmaps showing the spatial abundance of CancerCell and Immune cells predicted by Scaden and DestVI, respectively, in the mouse_PDAC dataset. The x and y axes represent the true spatial locations in the section as provided by the target PDAC dataset.
